# Supplementary figures and images for: Comparing feedback and spatial approaches to advance ecosystem-based fisheries management in a changing Antarctic
Source: PLoS One. 2020 Sep 8;15(9):e0231954. doi: 10.1371/journal.pone.0231954 (PMC7478840; doi:10.1371/journal.pone.0231954)

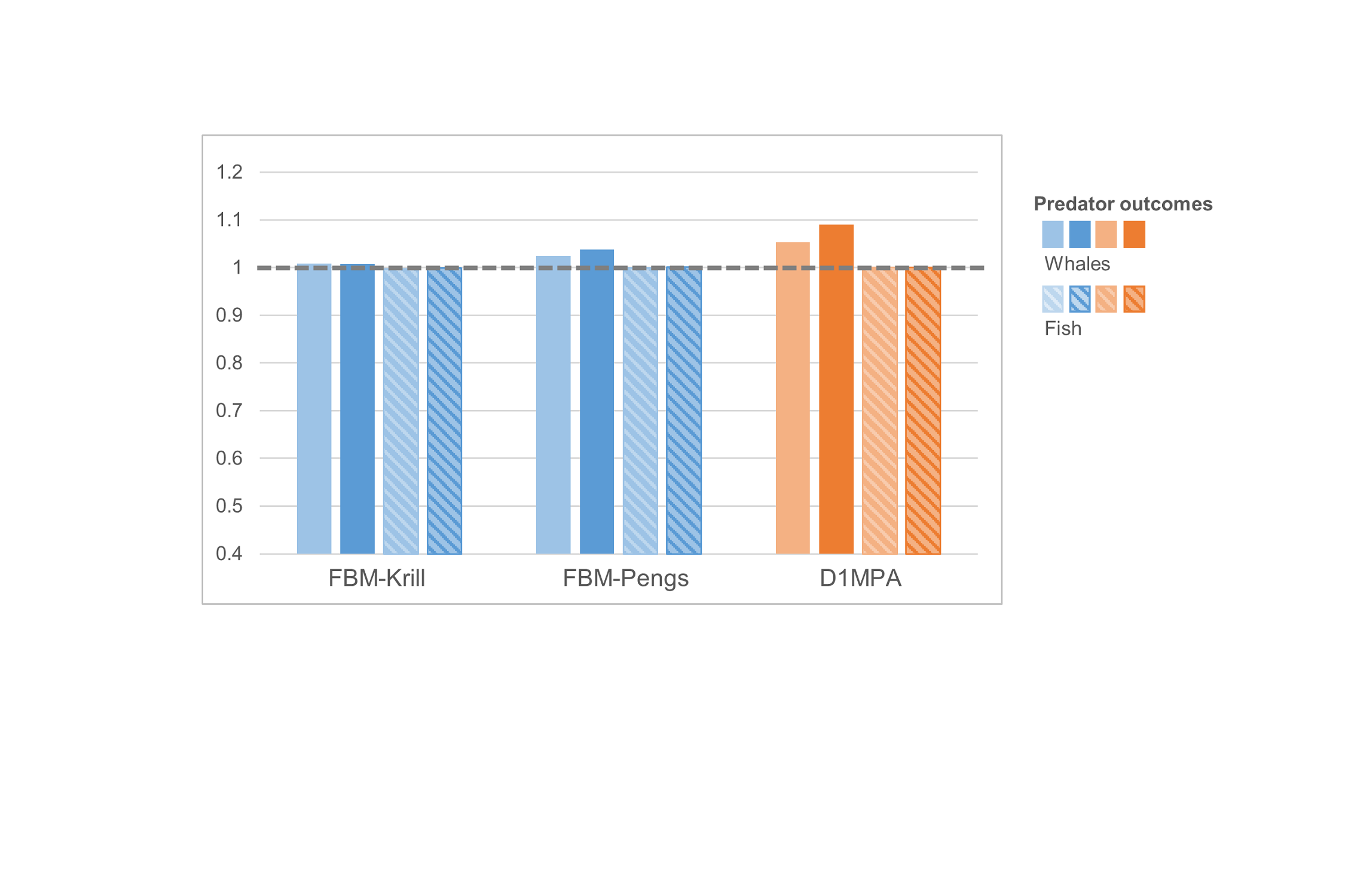

Supplement: S1 Fig — Lighter shades are at 30 years in the model run, and darker shades at 100 years. Feedback strategies are indicated in blue, and the MPA in orange. All results are referenced to the No FBM or No MPA scenarios, with the dashed grey line at 1.0 indicating no impact of FBM or the MPA. (TIF) [file pone.0231954.s001.tif]

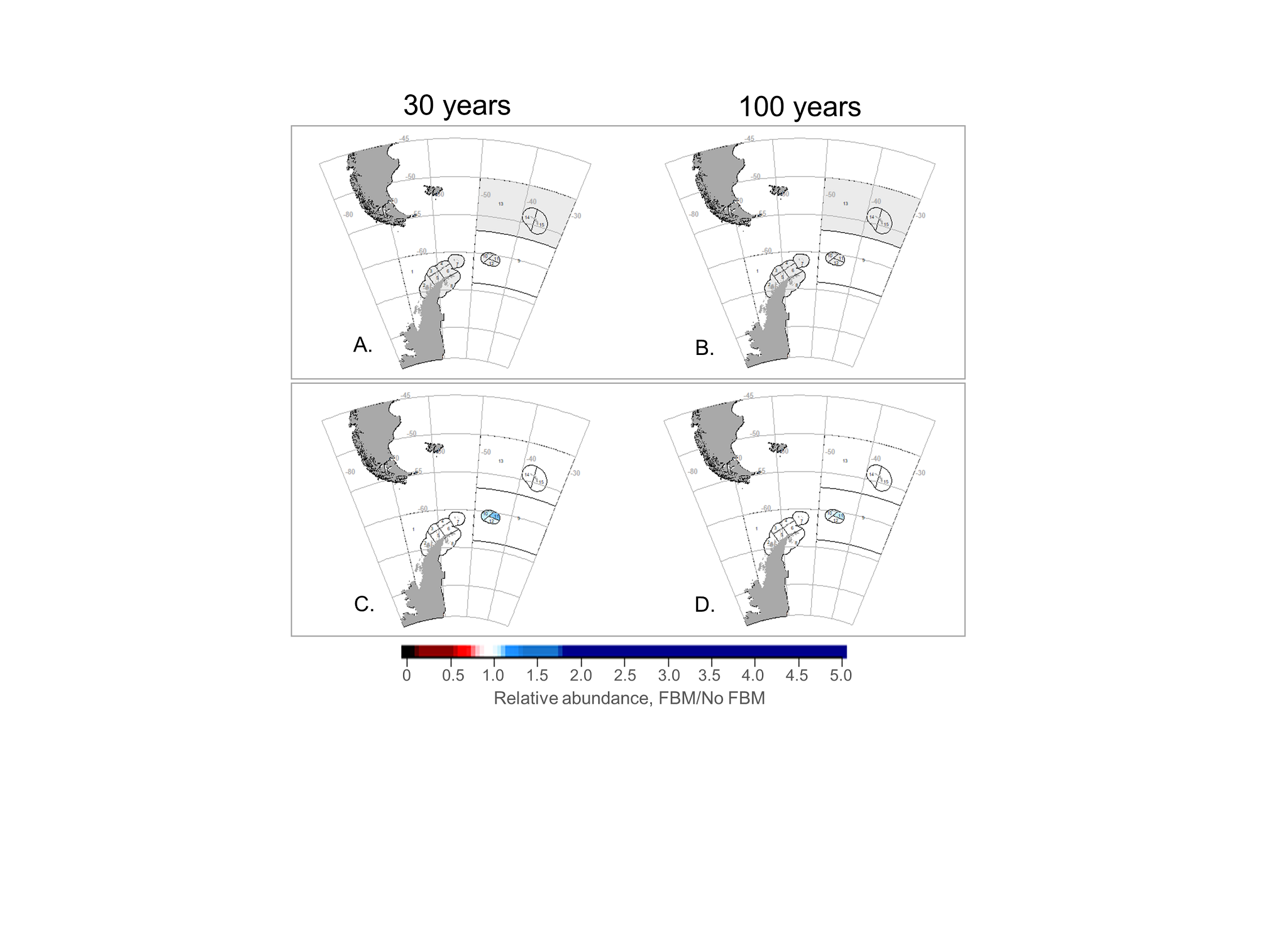

Supplement: S2 Fig — Projected whale (A, B) and fish (C, D) abundances given climate-change impacts on krill growth, with outcomes at 30 years in to the model run in the left column (A, C) and at 100 years in the right (B, D). Blues represent increases relative to the No FBM base case scenario and reds decreases; white and light colors indicate no or little change. Light grey denotes areas where the species group is not modeled. Note changes are relative to the No FBM base case within each SSMU, not the entire model arena. (TIF) [file pone.0231954.s002.tif]

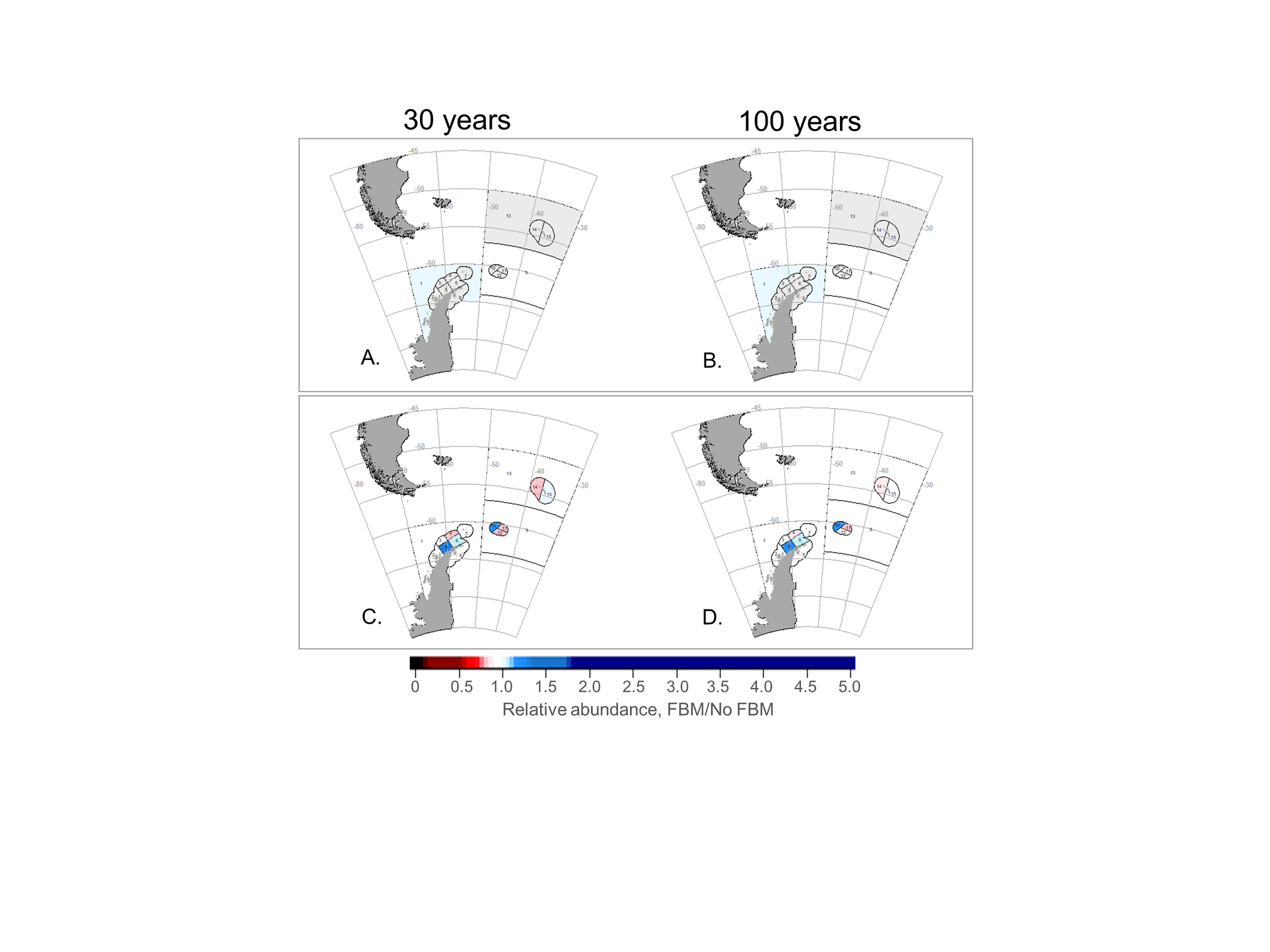

Supplement: S3 Fig — Projected whale (A, B) and fish (C, D) abundances given climate-change impacts on krill growth, with outcomes at 30 years in to the model run in the left column (A, C), and at 100 years in the right (B, D). All other details as in S2 Fig. (TIF) [file pone.0231954.s003.tif]

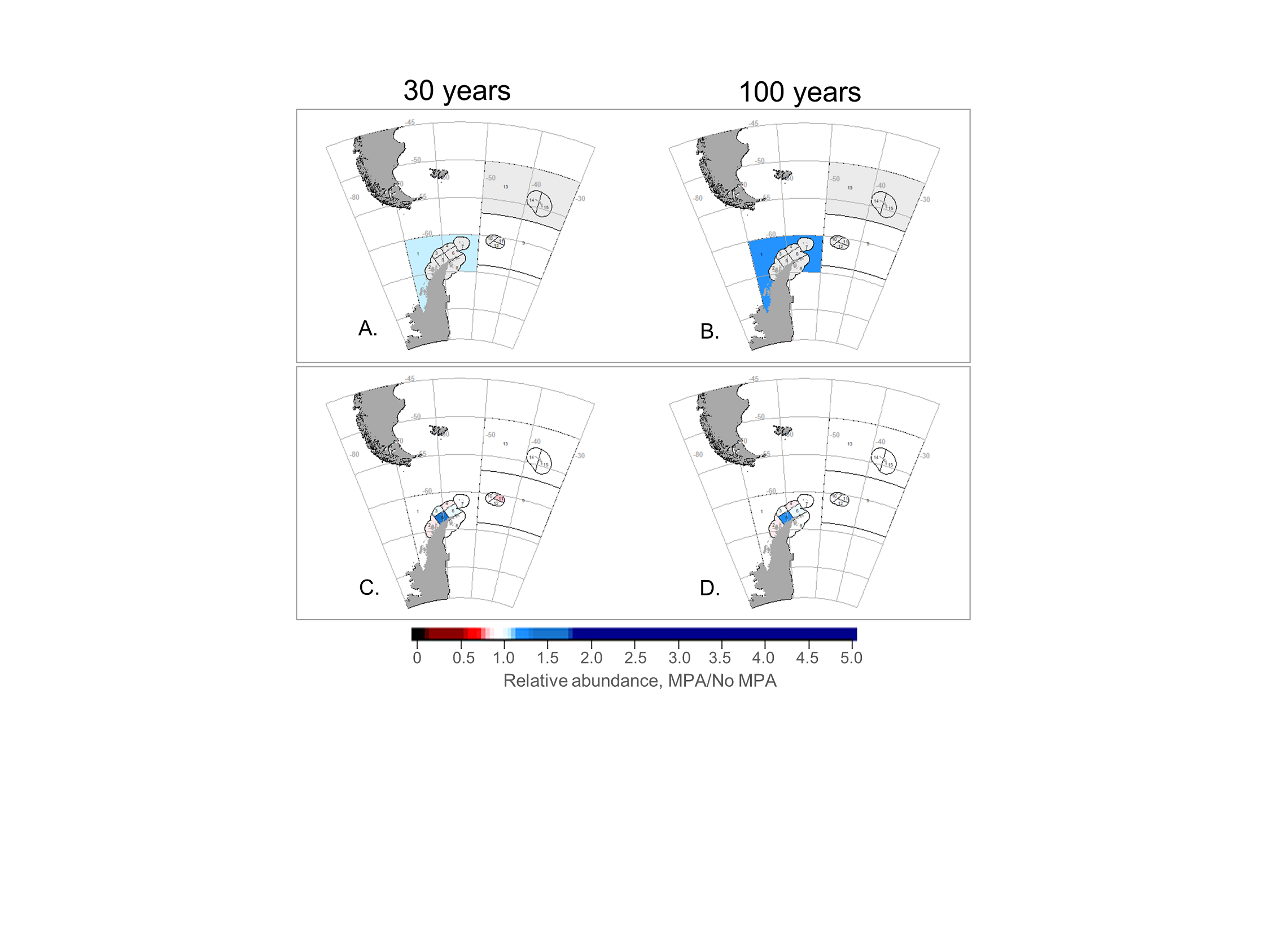

Supplement: S4 Fig — Projected whale (A, B) and fish (C, D) abundances given climate-change impacts on krill growth, with outcomes at 30 years in to the model run in the left column (A, C), and at 100 years in the right (B, D). All other details as in S2 Fig, aside from the base case being the No MPA scenario. (TIF) [file pone.0231954.s004.tif]

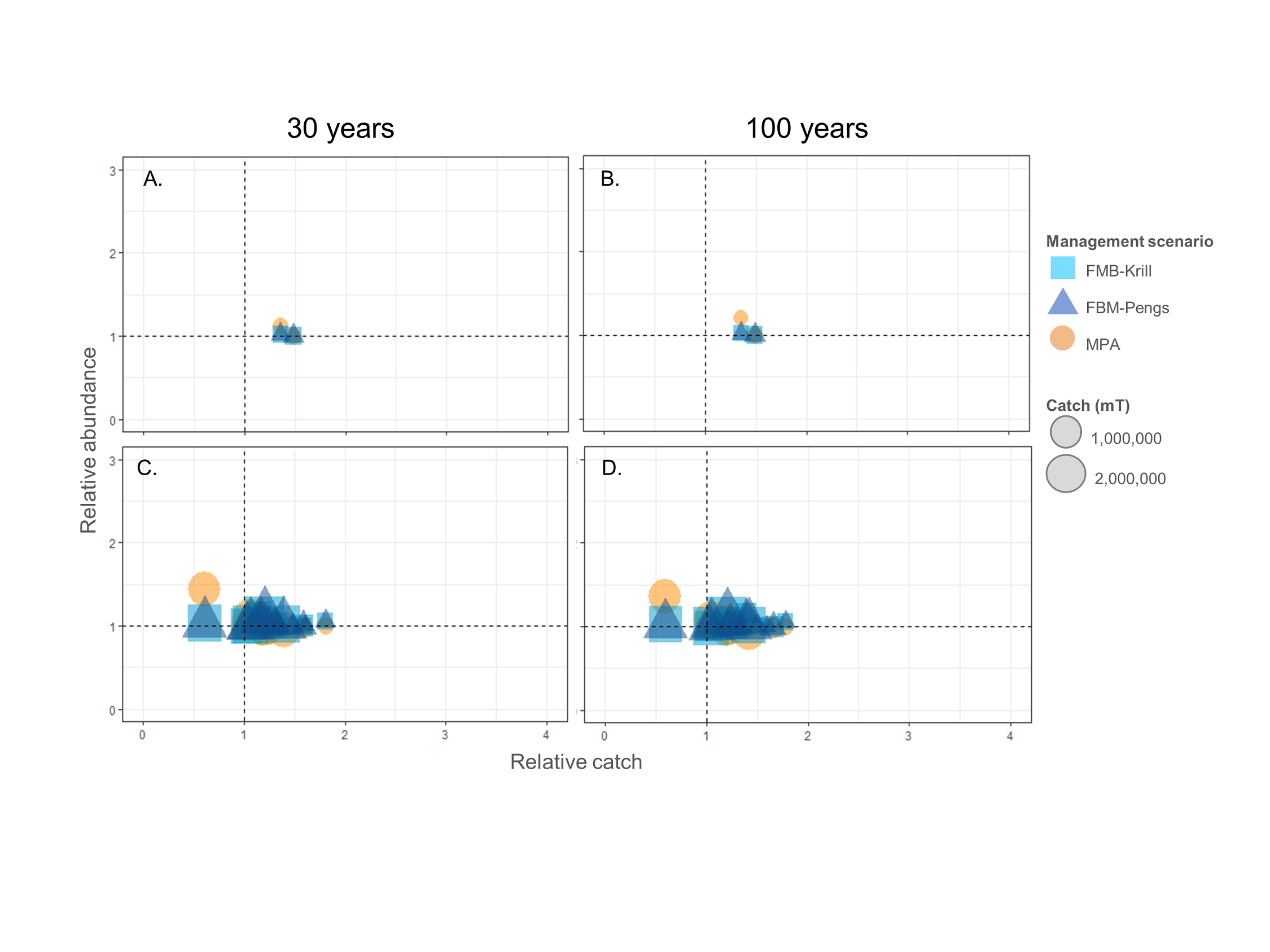

Supplement: S5 Fig — Relative catches (FBM/No FBM or MPA/No MPA, x-axis) and relative changes in the abundances (FBM/No FBM or MPA/No MPA, y-axis) of whales (A, B) and fish (C, D) given FBM-Krill (light blue squares), FBM-Pengs (dark blue triangles), and the MPA (orange circle) at 30 years (left column, A and C) and at 100 years (right column, B and D). The dashed lines represent no change in catch or abundance at x = 1 and y = 1, respectively. (TIF) [file pone.0231954.s005.tif]
