# Supplementary material for: Comparing feedback and spatial approaches to advance ecosystem-based fisheries management in a changing Antarctic
Source: PLoS One. 2020 Sep 8;15(9):e0231954. doi: 10.1371/journal.pone.0231954 (PMC7478840; doi:10.1371/journal.pone.0231954)
Supplement: S1 File — (DOCX) [file pone.0231954.s006.docx]

Comparing “feedback management” and marine protected areas to advance ecosystem-based approaches in a changing Antarctic

**Short title:** Comparing feedback management and marine protected areas in the Antarctic.

Emily S. Klein and George M. Watters

## Supporting Information

### Additional model information

A both useful and challenging aspect of ecosystem models is their complex nature, involving numerous equations, parameters, and input data. The spatially-explicit, dynamic Krill Predator Fisher Model (KPFM2) we use here is no exception. KPFM2’s extensive equations and parameters are outlined in detail in Watters et al. [1] and included Appendices, and while we cannot duplicate that work here, we instead provide a summary of model parameters and equations in the S1 and S2 Tables. Further information on the MPA partitioning in the model is provided in Klein and Watters [2]

The detailed parameters and equations [1] are coded into the core KPFM2 model via the R language [3] or as model inputs. This code and all relevant inputs, along with detailed READ ME files for its implementation, are available online at <https://github.com/EmilyKlein/KPFM2/> [4]. To implement KPFM2 for scenario exploration, as here, these scenarios involve additional R code to run either a marine protected area (MPA) or feedback management (FBM), available at <https://github.com/EmilyKlein/KPFM2_MPA_FBM> [5]. These code and inputs are made available so that others may reproduce and build on our work, but are not to serve as an explanation or reasoning behind the ecosystem model itself. We strongly encourage interested readers to first refer to the substantial text in Watters et al. [1], which provides the necessary details in terms of explanation and rationale for KPFM2. We then recommend an interested user familiarize themselves with the KPFM2 model [4] before the additional scenario exploration, although all necessary code and inputs to run MPA and FBM scenarios is included at [5].

Finally, we have included all of the results used to create the figures in the manuscript as Supporting Information as well (S3 Table, an external Excel spreadsheet).

### Results for additional predator groups

We assessed outcomes of the MPA for all krill-dependent predator groups in our ecosystem model, with results for those predator groups beyond penguins and seals provided here (Fig S1-S4).

### References

1. Watters GM, Hill SL, Hinke JT, Matthews J, Reid K. Decision-making for ecosystem-based management: evaluating options for a krill fishery with an ecosystem dynamics model. Ecol App. 2013;23(4): 710-725. doi: 10.1890/12-1371.1.
2. Klein ES, Watters GM. What’s the catch? Profiling the benefits and costs associated with marine protected areas and displaced fishing in the Scotia Sea. PLoS One.
3. R Core Team. R: A language and environment for statistical computing. R Foundation for Statistical Computing. Vienna, Austria; 2017. <http://www.R-project.org/>.
4. Klein ES, Hinke JT, Watters GM. KPFM2: Krill-Predator-Fishery Model; 2019 [cited 29 Nov 2019]. Model code and input files [Internet]. Available from: <https://github.com/EmilyKlein/KPFM2>.
5. Klein ES, Hinke JT, Watters GM. KPFM2: Krill-Predator-Fishery Model – MPA and FBM extensions; 2019 [cited 29 Nov 2019]. Model code and input files [Internet]. Available from: <https://github.com/EmilyKlein/KPFM2>_MPA_FBM.

### Tables and figures

**S1 Table**. **Model parameters and state variables in KPFM2**.

| **Parameter or Variable** | **Description** |
| --- | --- |
| *Predators* | |
| $\bar{M}_{k,i,s}$ | Mean instantaneous rate of natural mortality |
| $\upsilon_{k,i,s}$ | Level of foraging performance that distinguishes a good year from a bad year |
| $z_{k,i,s}$ | Proportion of potential variation in survival that is explained by variations in mean per-capita foraging performance |
| $\rho'_{k,i}$ | Age at recruitment to adult stage |
| $\alpha'_{k,i,s}$ | Maximum per-capita recruitment at low adult abundance when all adults breed |
| $Rpeak_{k,i,s}$ | Maximum recruitment when all adults breed |
| $Speak_{k,i,s}$ | Adult abundance that produces maximum recruitment |
| $\varphi'_{k,i,s}$ | Shape parameter determining ratio of effective breeder abundance to adult abundance |
| $Q_{k,i,s}^{*}$ | Maximum per-capita potential consumption |
| *Jphi* | Shape parameter that scales potential recruitment based on mean per-capita foraging success during first winter of life |
| $\kappa_{k,i,s}$ | Krill density at which predators achieve half of maximum potential per-capita consumption |
| $q_{k,i,s}$ | Functional response shape parameter |
| $p_{k,i\leftarrow j,s}$ | Proportion of krill-derived energy that predators breeding in SSMU *i* obtain from SSMU *j* |
| $c_{k,i}$ | Competitive strength of predators relative to that of the fishery |
| $\Omega_{k,i,0}$ | Initial potential demand for krill by a single predator stock breeding in SSMU *i* |
| $P_{k,i,0}$ | Initial abundance of predators that breed in SSMU *i* |
| init.type | Specifies whether initial abundance is input directly or determined from consumption |
| *Krill* | |
| *$v_{i\to j,s}$* | Instantaneous rate of krill movement from area *i* to area *j;* rates are set as a proportion of the overall krill that move from SSMU *i* to SSMU *j* in season *s.* |
| $K_{i,t}$ | Abundance of krill |
| *$M0_{i,s}$* | Instantaneous rate of natural, non-predation mortality |
| *$\alpha_{i,s}$* | Maximum recruitment |
| *$\beta_{i,s}$* | Adult abundance that produces half of maximum recruitment |
| $\rho$ | Age at recruitment to adult stage |
| $\varphi_{i,s}$ | Scalar that mediates environmental effects on krill recruitment |
| $X_{t}$ | Environmental index influencing recruitment |
| *$\sigma^{2}$* | Process variance in ln(recruitment) |
| $\delta_{i}$ | Fraction of abundance available for harvest and predation |
| *$K_{i,0}/A_{i}$* | Initial density of krill |
| *$\bar{w}_{i}$* | Average weight of an individual krill |
| *Fishery* | |
| $C_{H,i}$ | Historical catch of krill |
| $A_{i}$ | Area of the SSMU |
| $\gamma$ | Overall harvest rate |
| $L$ | Precautionary catch limit |
| $\eta_{i}$ | Threshold krill density (g ·m^-2^) that sets $C_{i,t}=F_{i,t}=0$ |
| $f_{i,s}$ | Proportion of allocated catch taken in season *s* |
| $c_{F,i}$ | Competitive strength of fishery relative to those of predators |

Description of the parameters in KPFM2 (adopted from Tables B2 and B3, Watters et al. 2013).

**S2 Table. Basic equations for the implementation of KPFM2 used in this work.**

| **Krill** | |
| --- | --- |
| Abundance of krill | 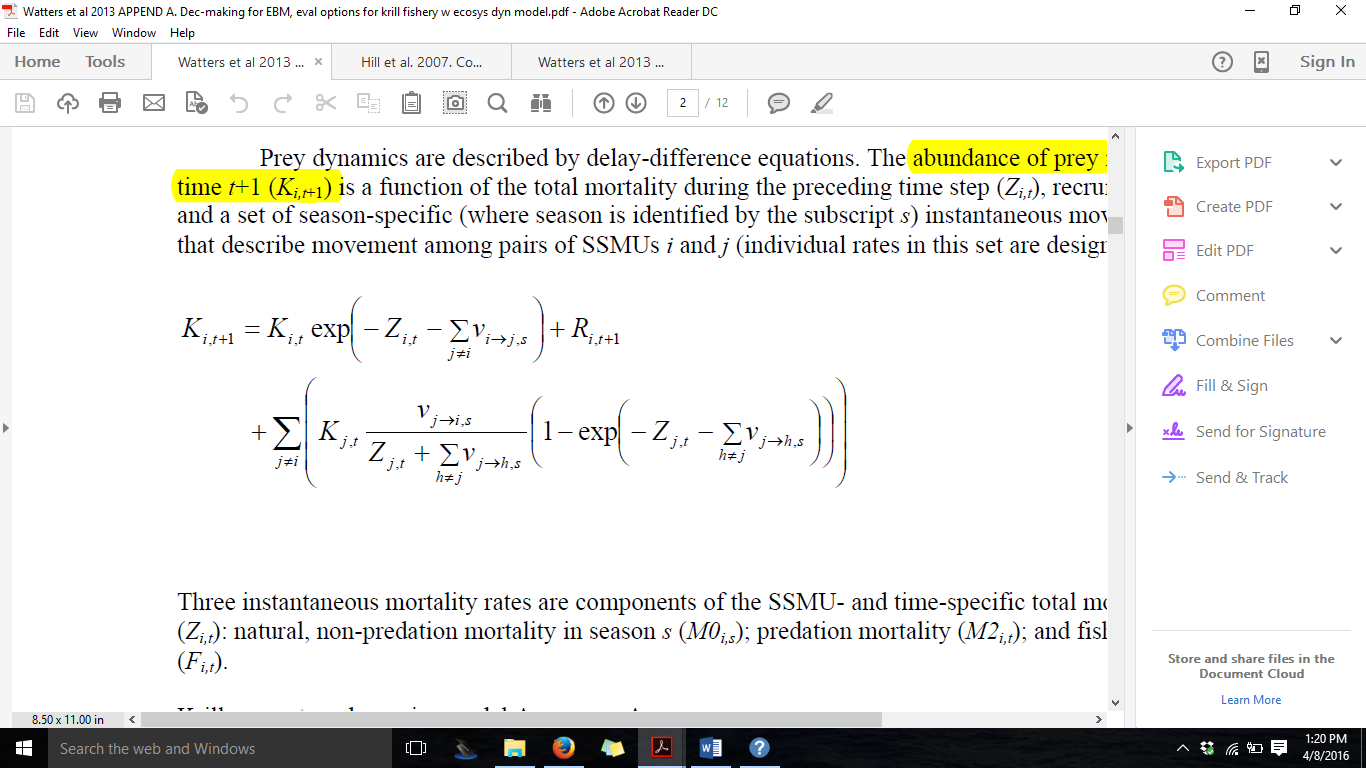 |
| Mortality of krill | 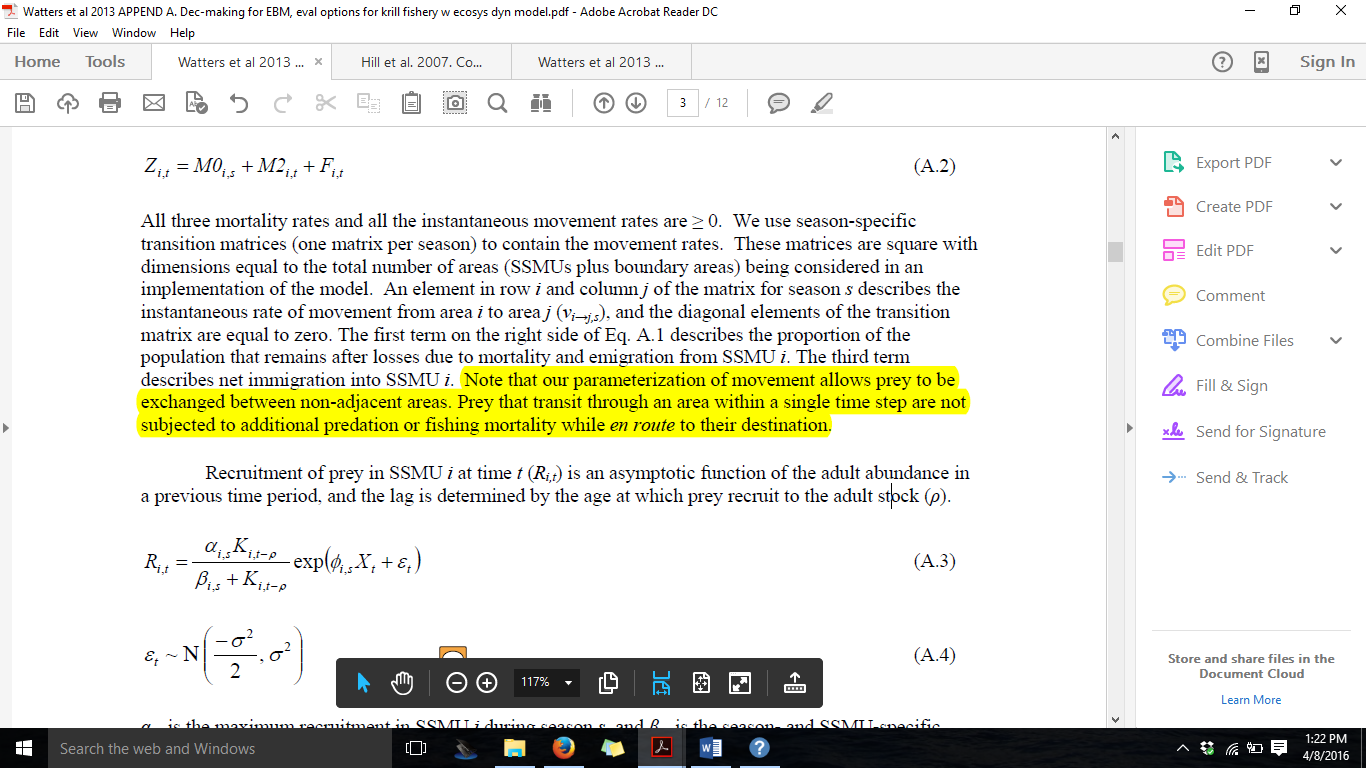 |
| Krill recruitment | 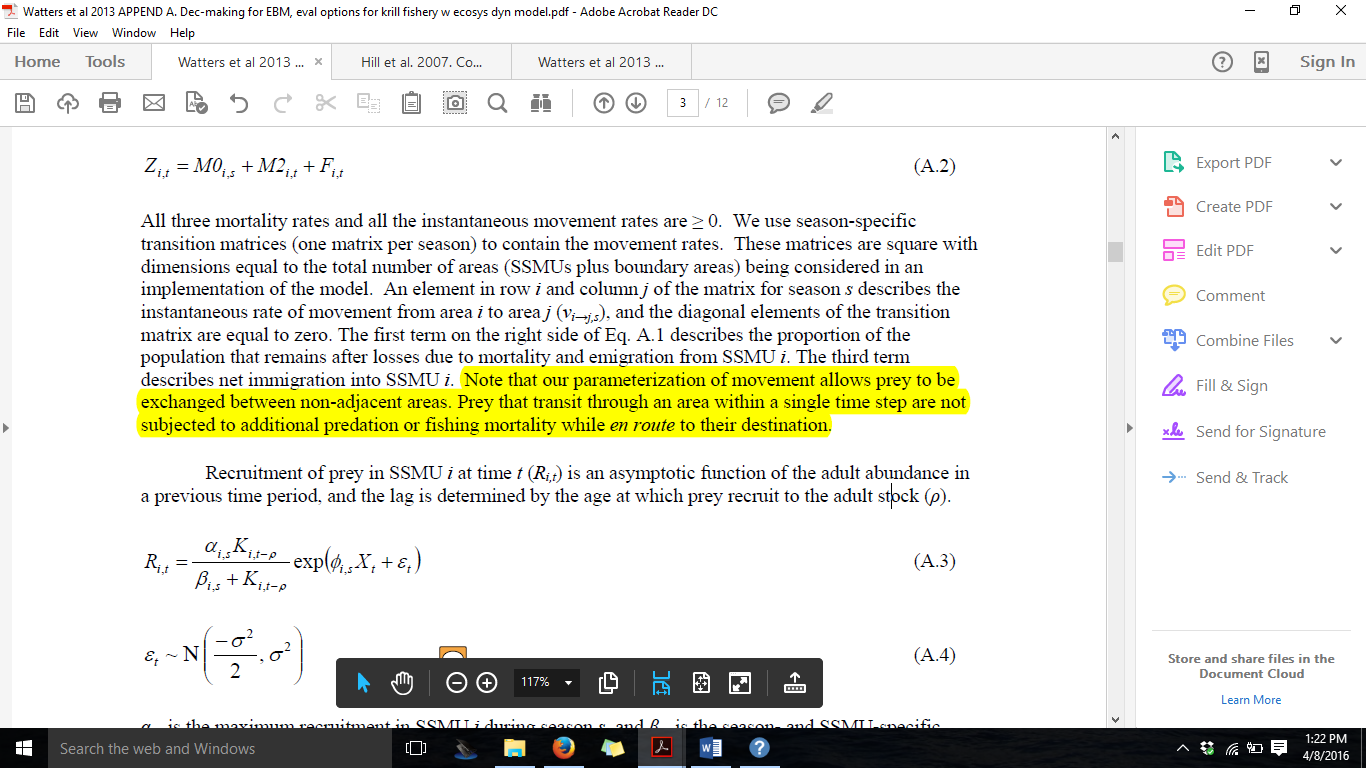 |
| Error term used to vary krill recruitment | 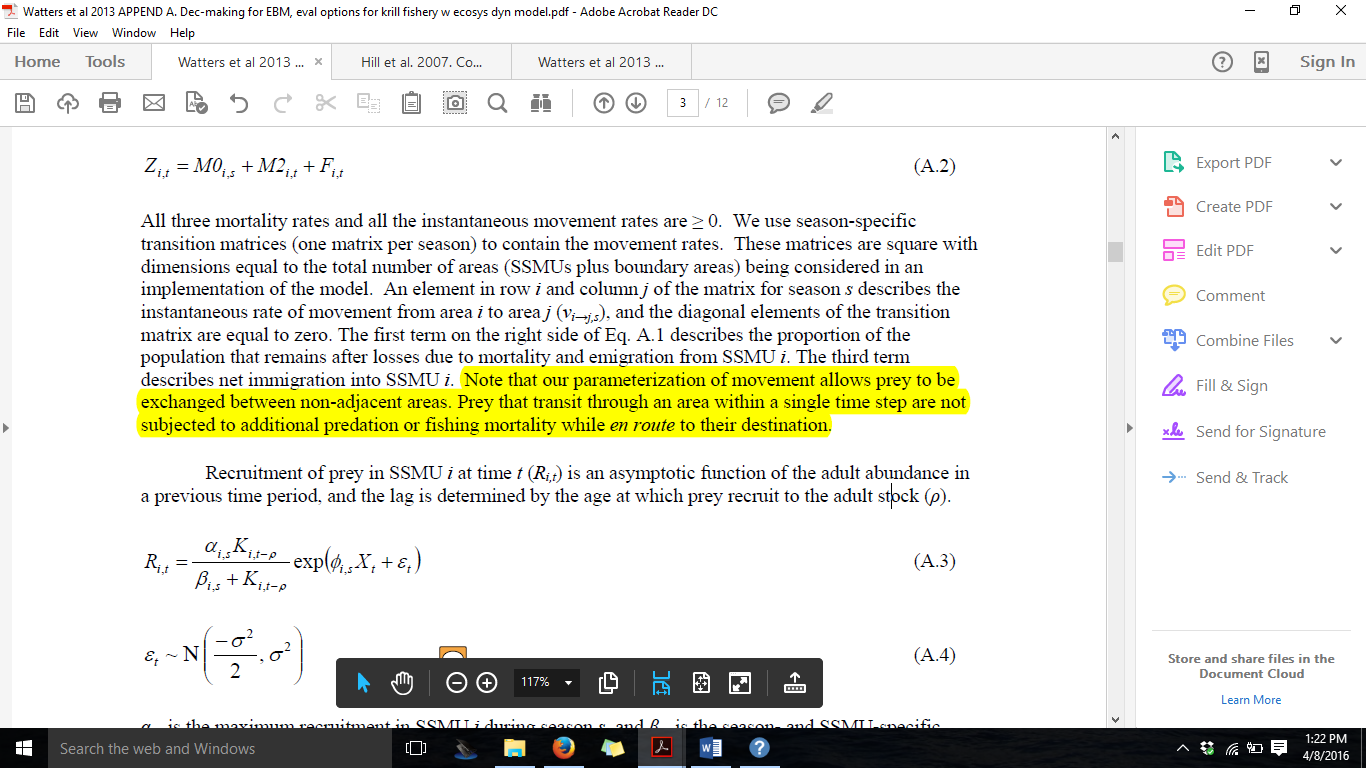 |
| **Predators** |  |
| Predator potential per-capita consumption | 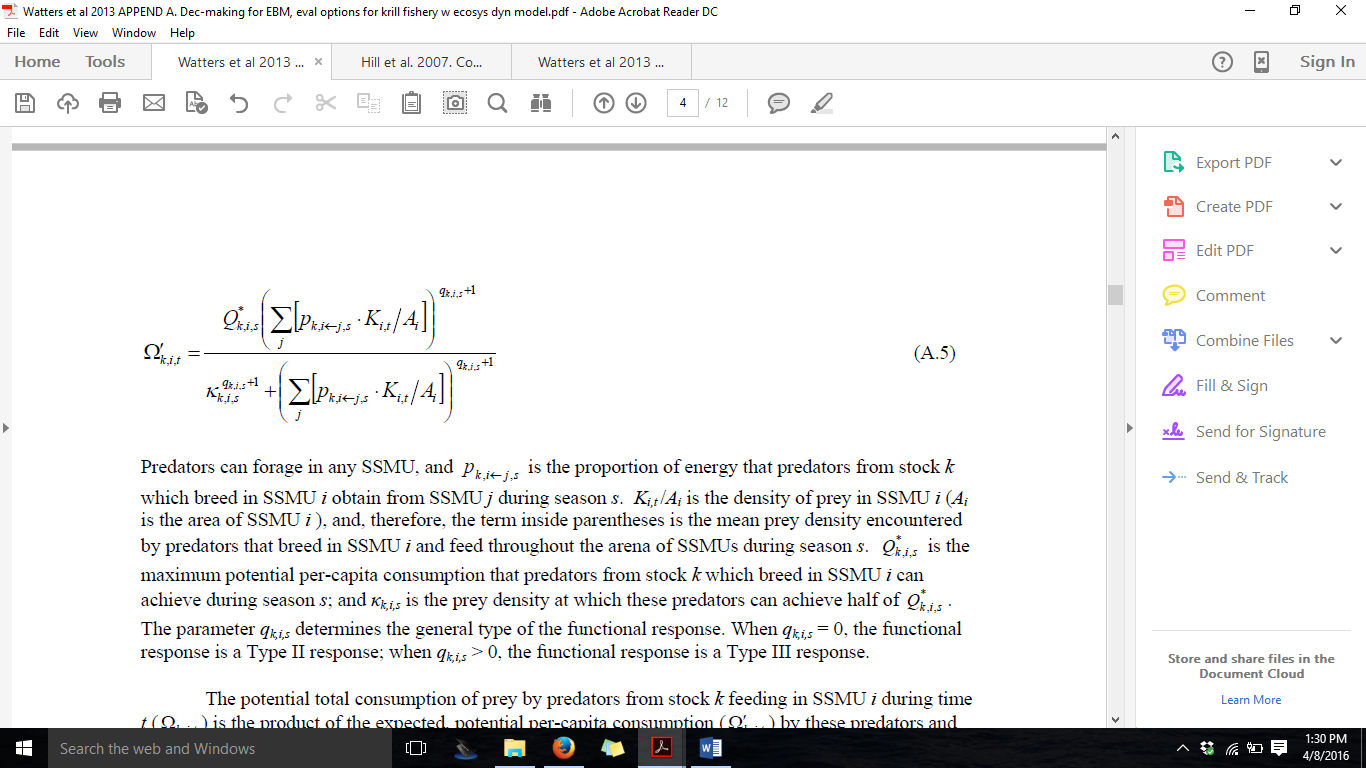 |
| Predator total potential consumption | 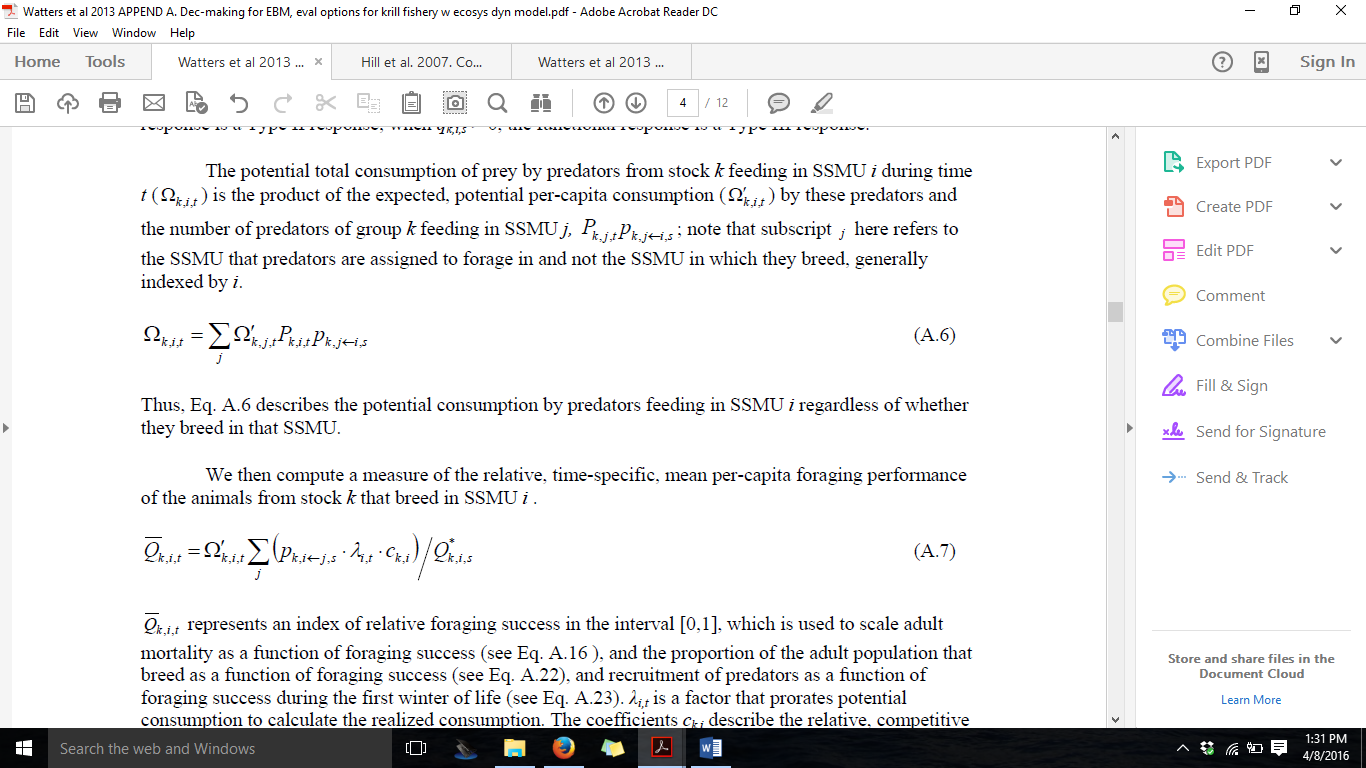 |
| Mean per capita predator foraging performance | 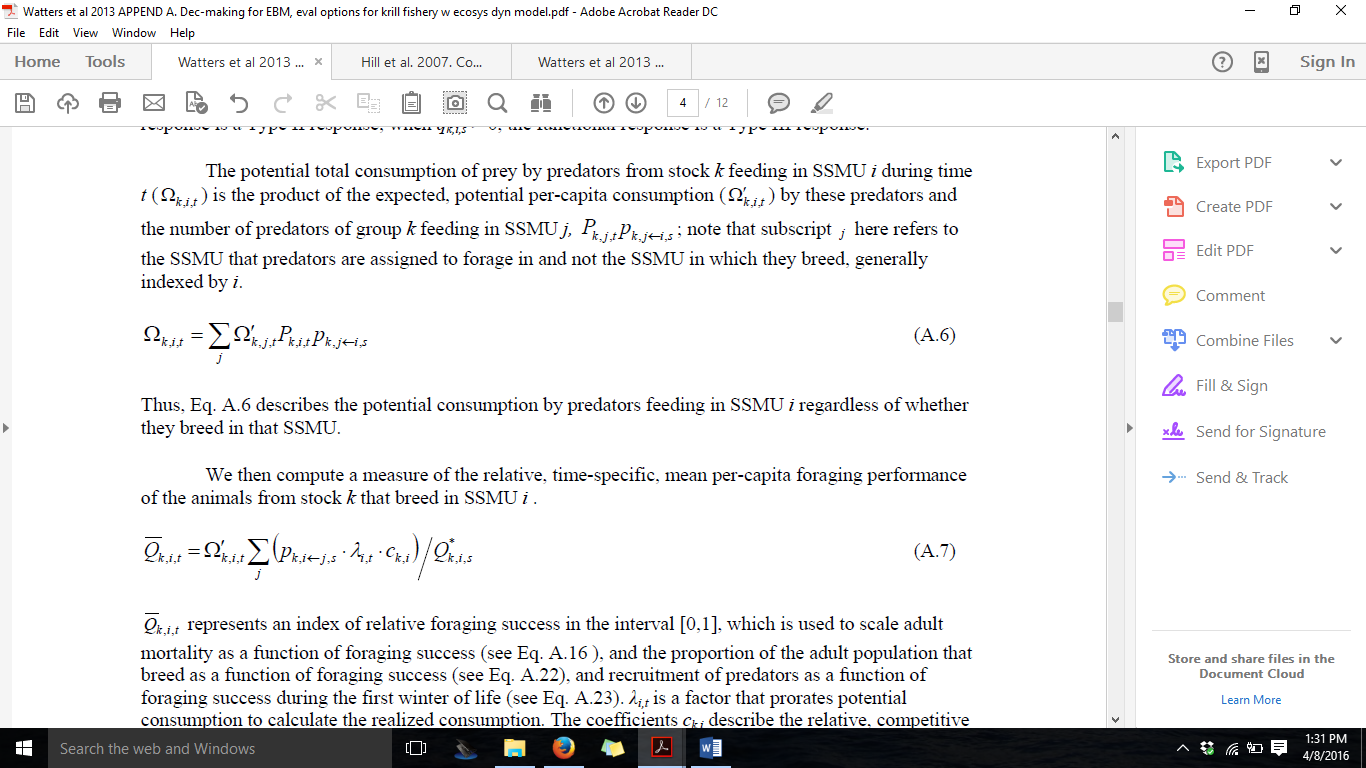 |
| Predator abundance | 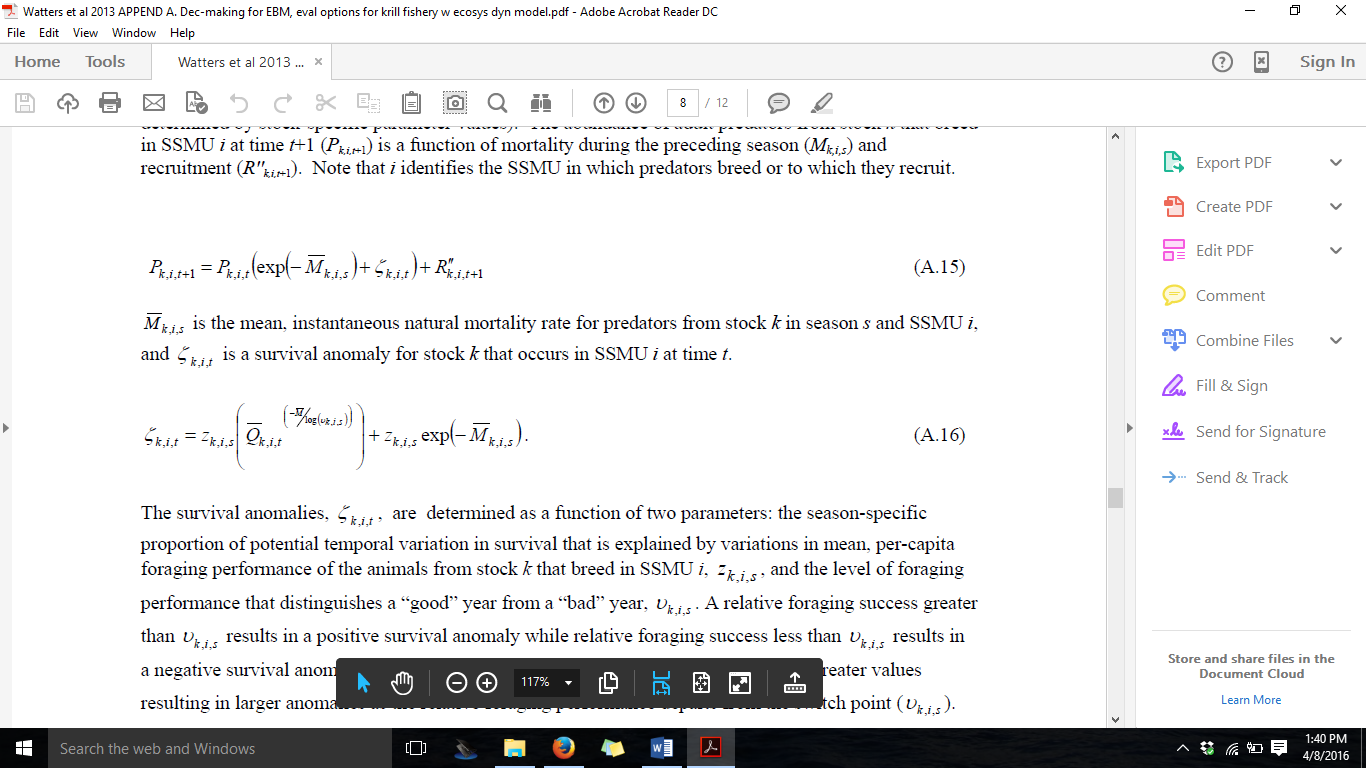 |
| Survival anomaly | 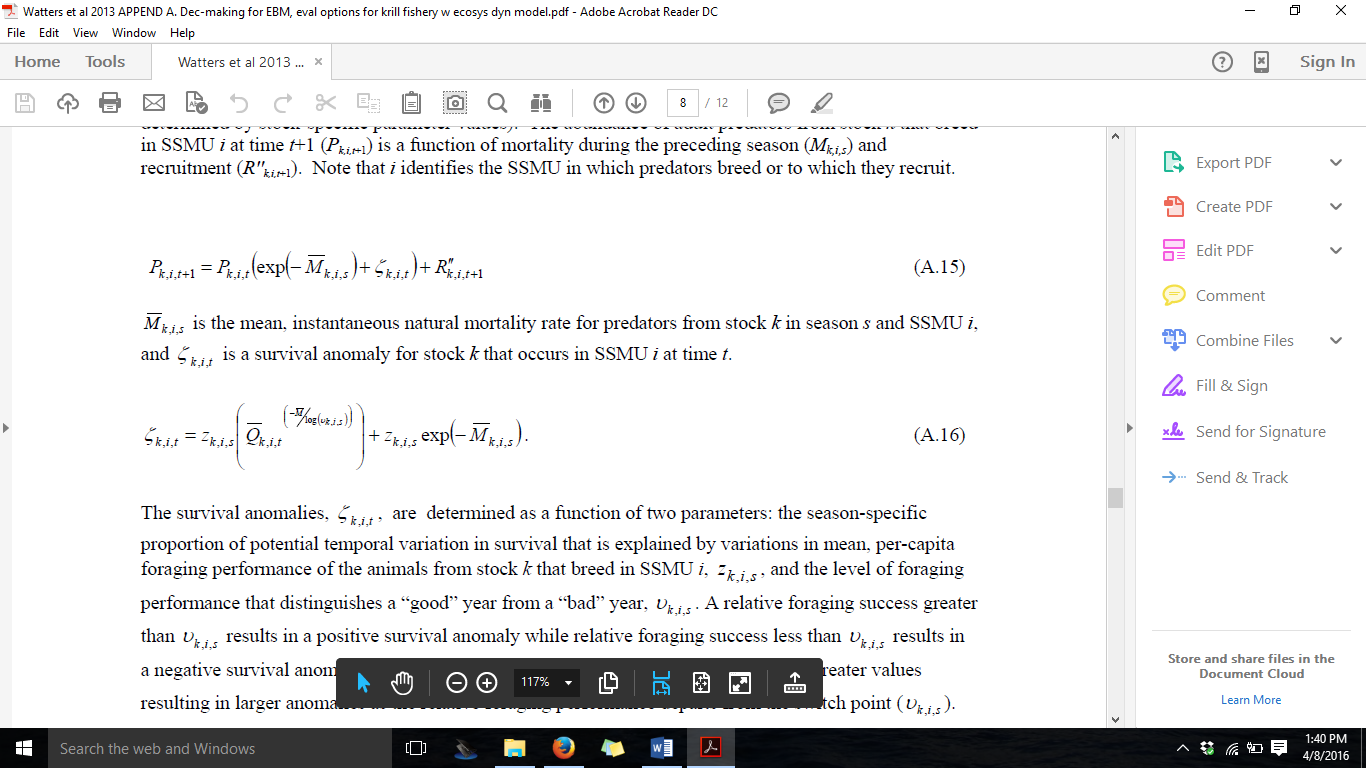 |
| Predator recruitment | 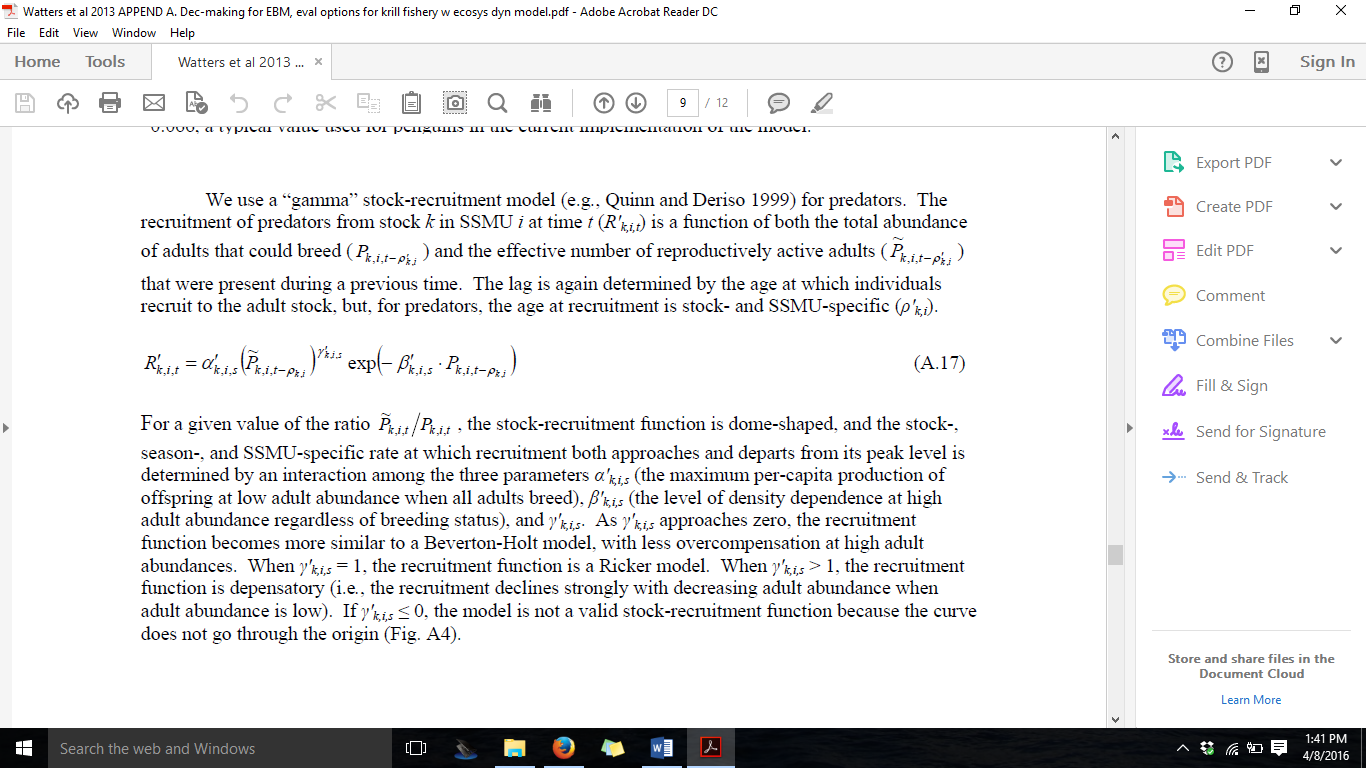 |
| Maximum recruitment when all predator adults breed | 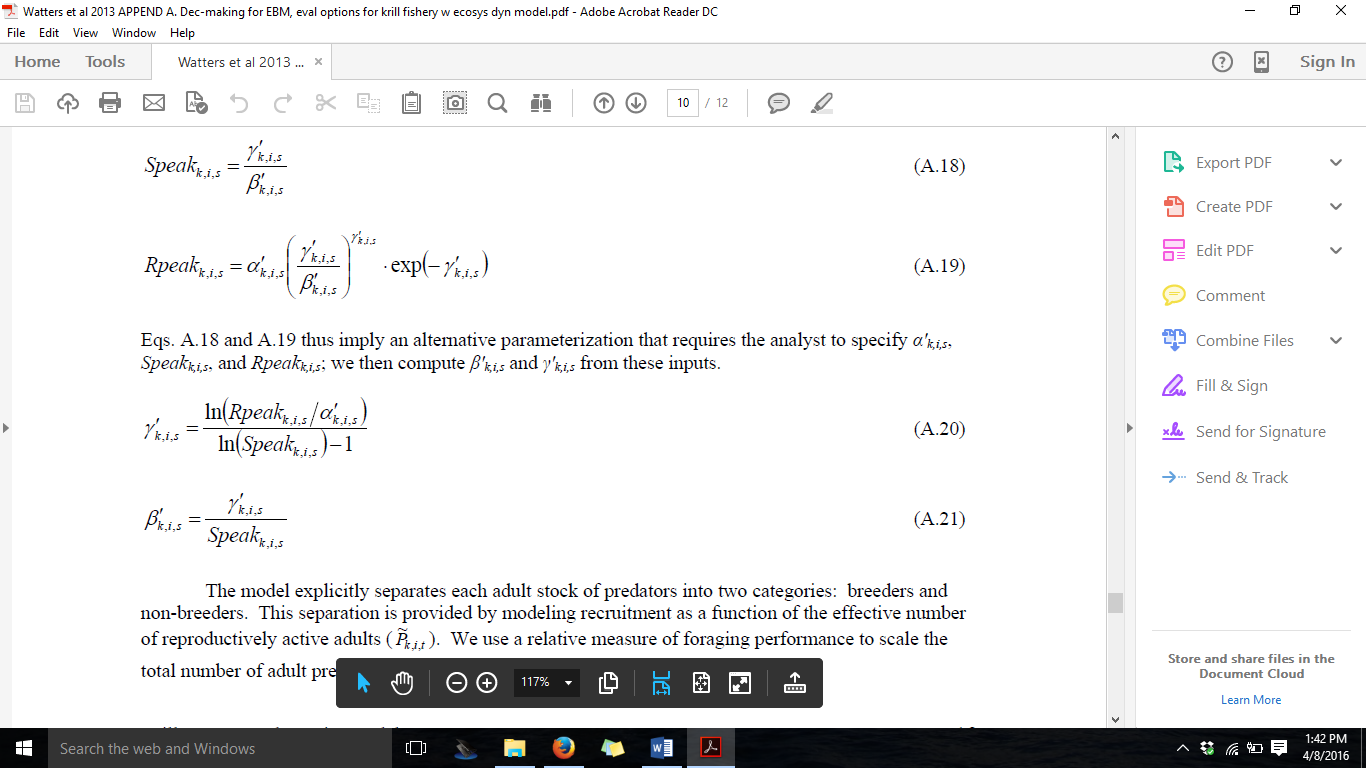 |
| Adult abundance that produces maximum recruitment | 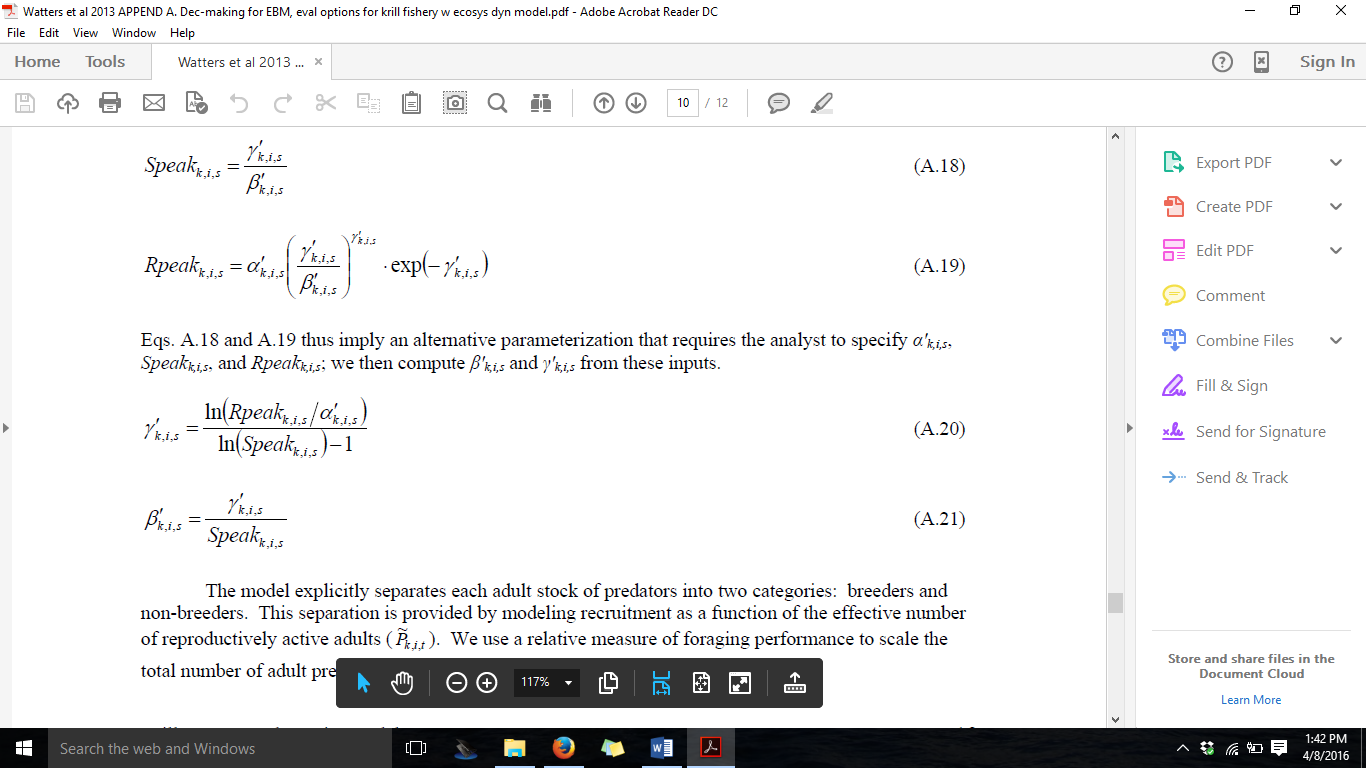 |
| Level of density dependence at high predator adult abundance regardless of breeding status | 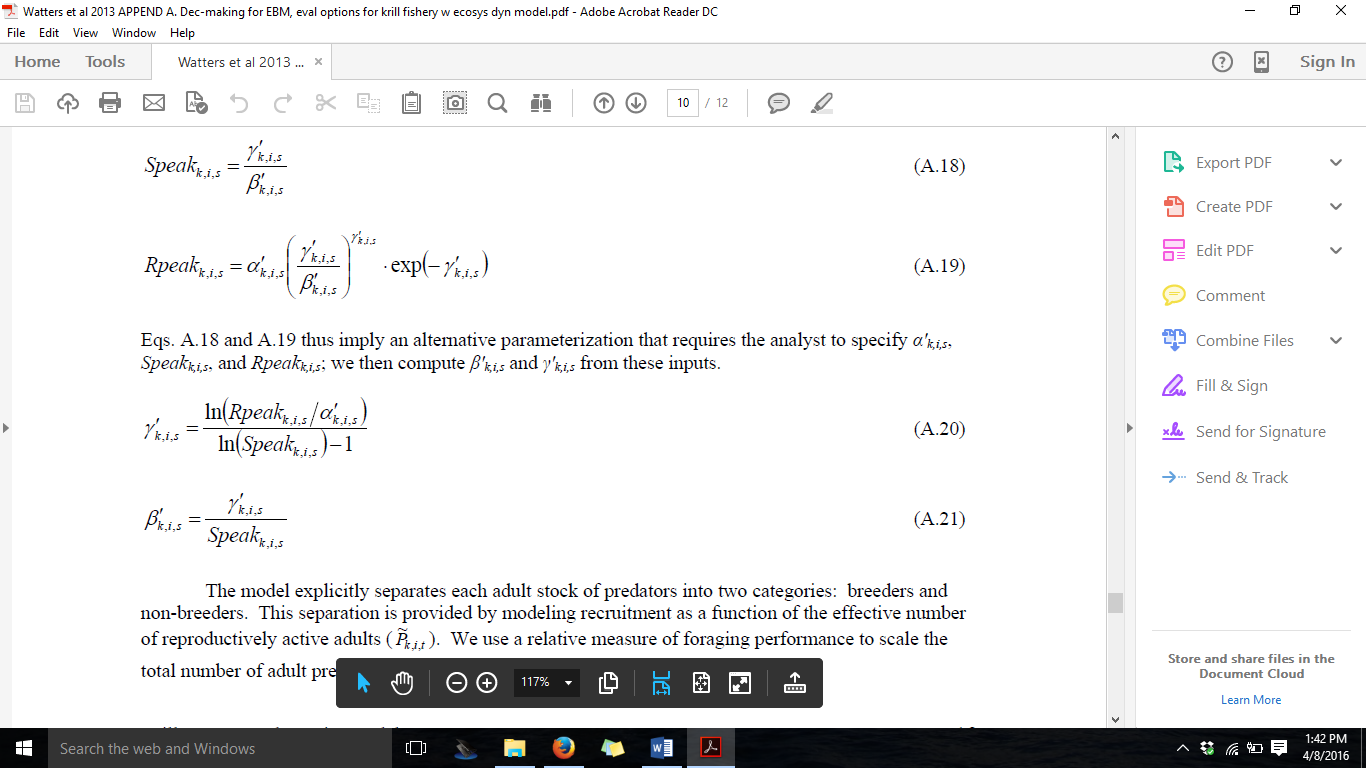 |
| Control of recruitment function: as *γ'k,i,s* approaches zero, recruitment approaches Beverton-Holt model, at 1 it is a Ricker model, at >1 recruitment is depensatory (at low adult abundance, recruitment declines strongly with decreasing adult abundance). | 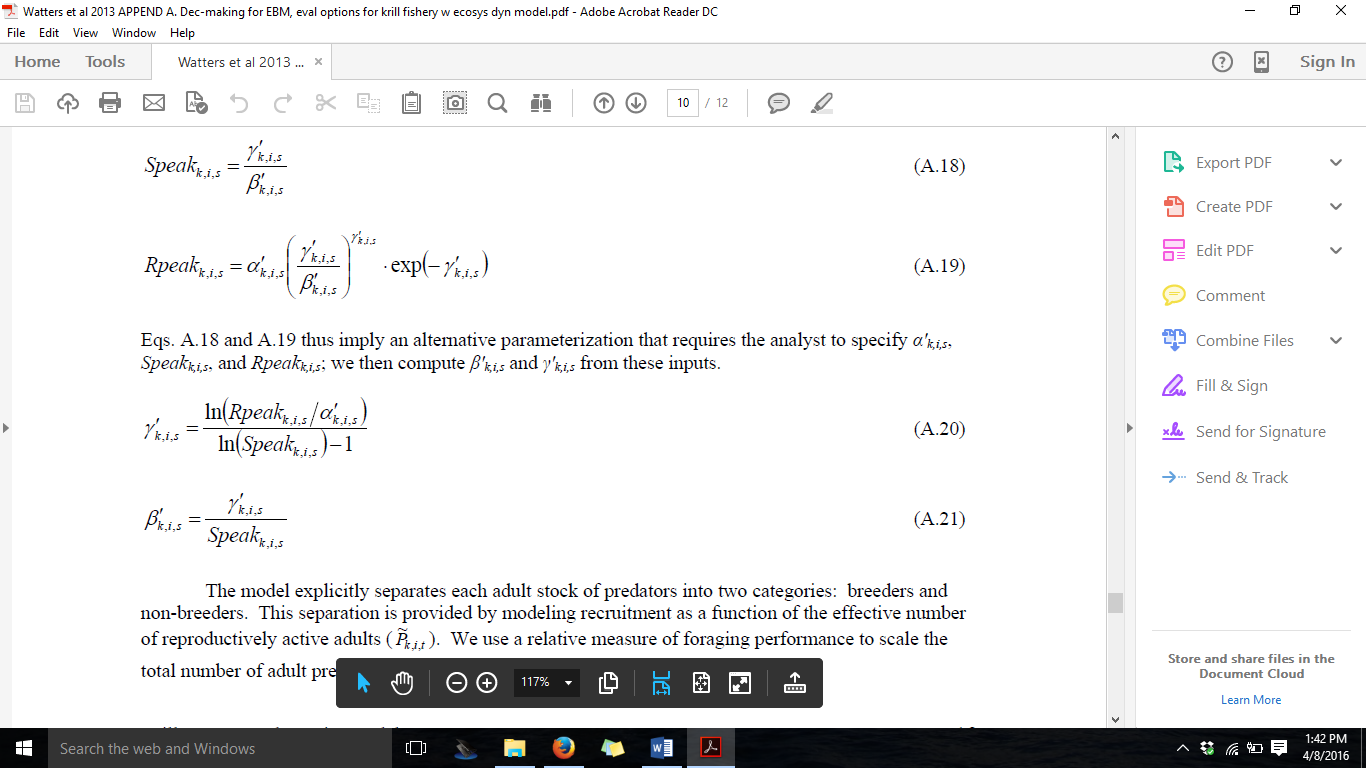 |
| Effective number of reproductively active adults | 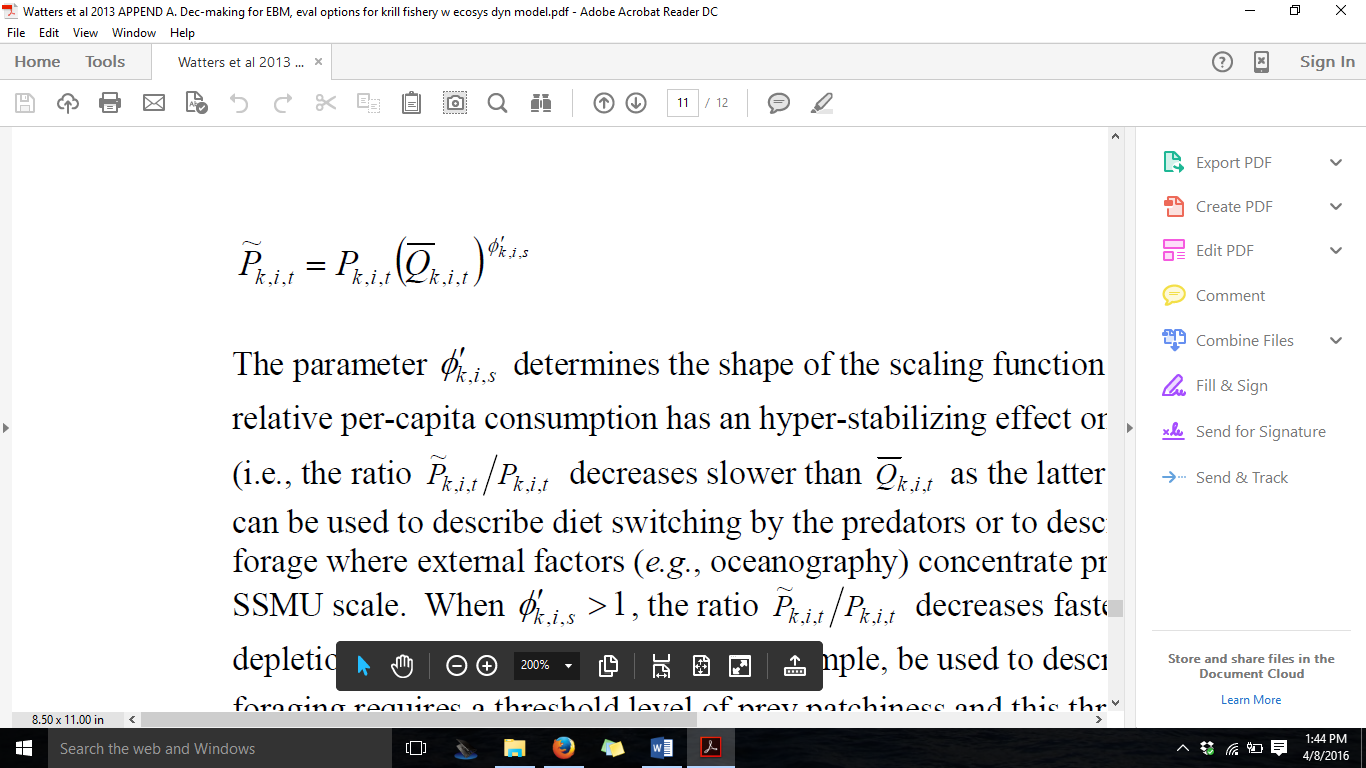 |
| **Fishery** |  |
| Allocated catch | 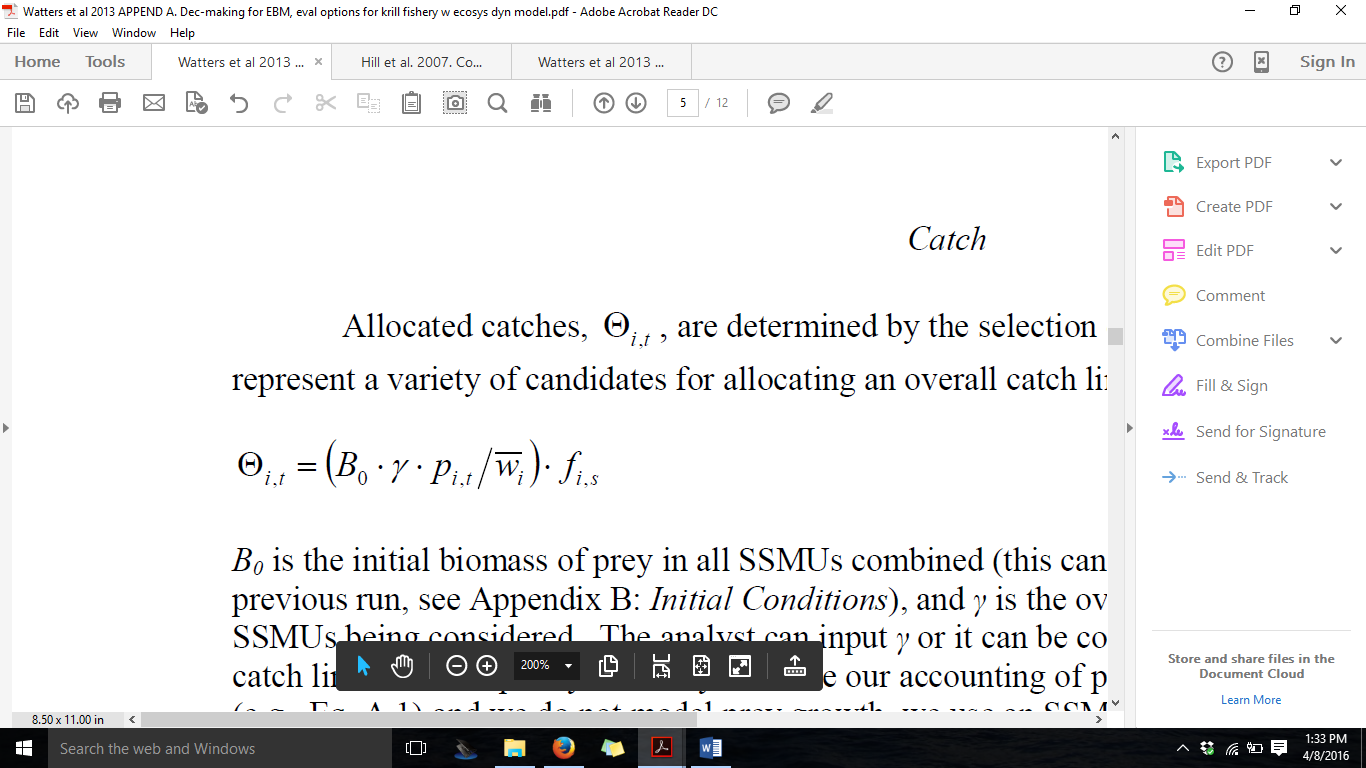 |

See S1 Table for parameter descriptions; adopted from Watters et al. [1] Appendix A.


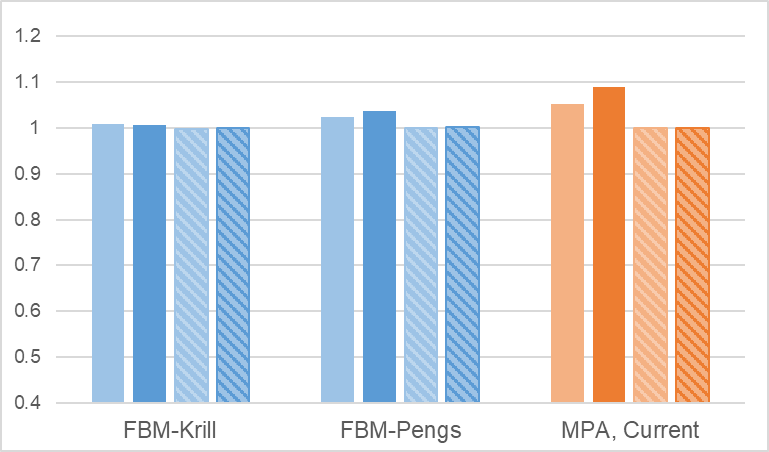


**Predator outcomes**

Whales

Fish

A.

**Fig S1. Relative total changes in the abundance of additional predator groups across modeled scenarios and aggregated at the scale of the full model arena**. Lighter shades are at 30 years in the model run, and darker shades at 100 years. Feedback strategies are indicated in blue, and the MPA in orange. All results are relative to the No FBM or No MPA scenarios, with the dashed grey line at 1.0 indicating no impact of FBM or the MPA.

B.


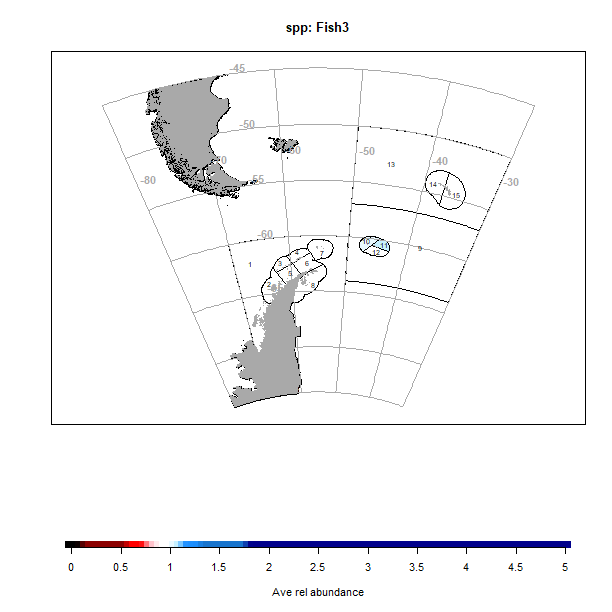

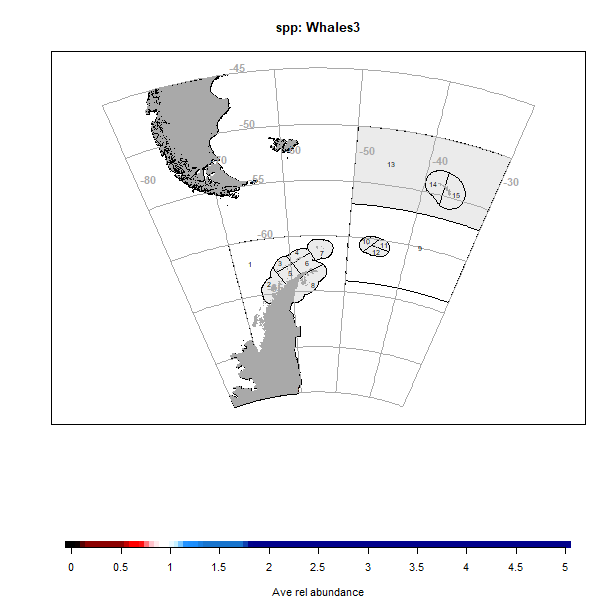

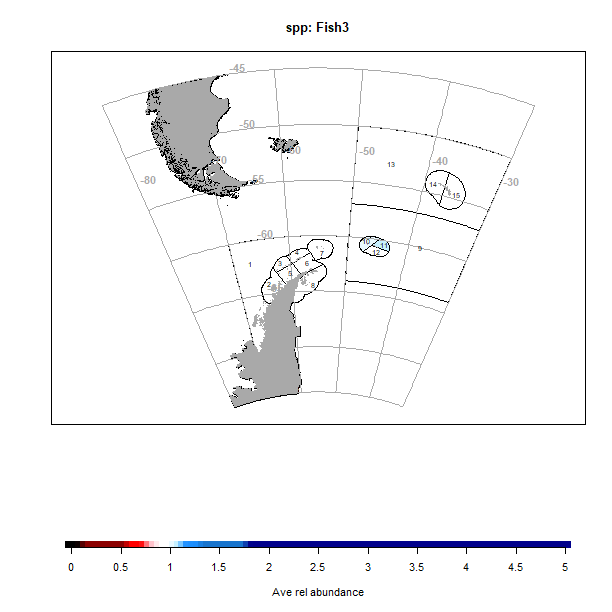

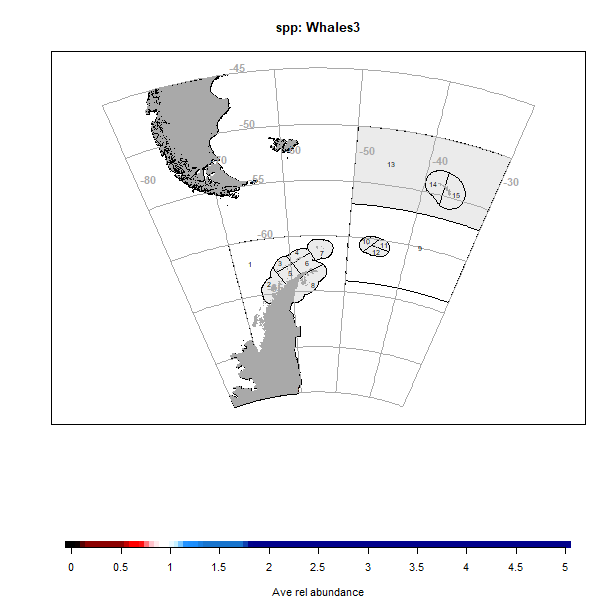

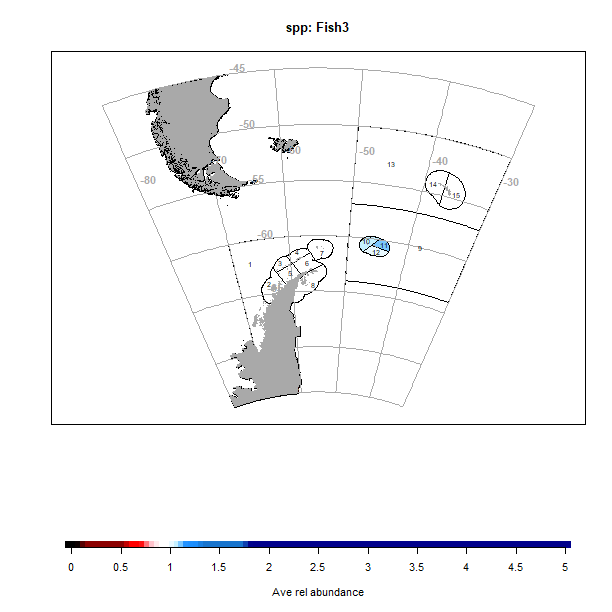


B.

C.

D.

A.

Relative abundance, FBM/No FBM

**Fig S2**. **SSMU-specific** **outcomes of FBM-Krill for additional predator groups under a modeled climate change impact**. Projected whale (A, B) and fish (C, D) abundances given climate-change impacts on krill growth, with outcomes at 30 years in to the model run in the left column (A, C) and at 100 years in the right (B, D). Blues represent increases relative to the No FBM reference scenario and reds decreases; white and light colors indicate no or little change. Light grey denotes areas where the species group is not modeled as resident. Note changes are relative to the No FBM reference within each SSMU, not the entire model arena.


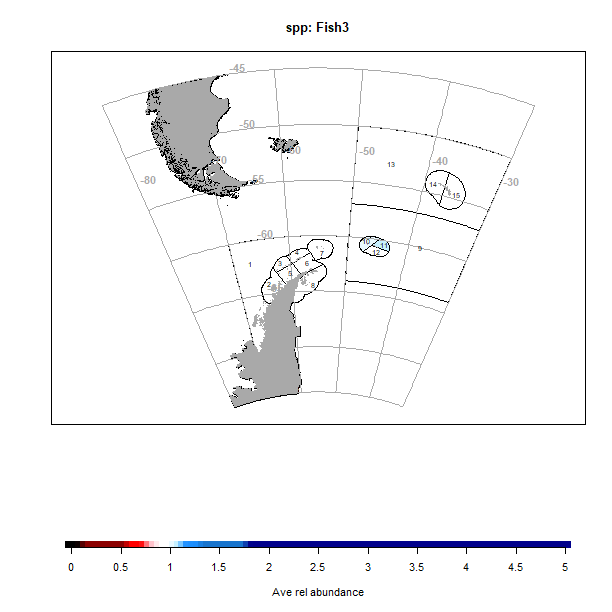

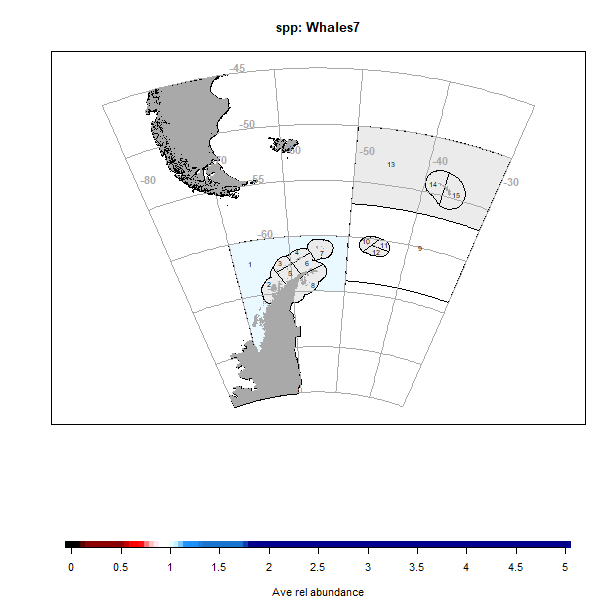

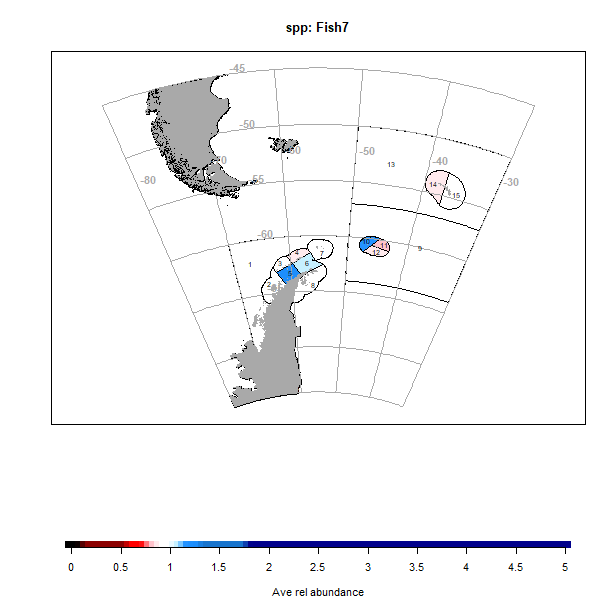

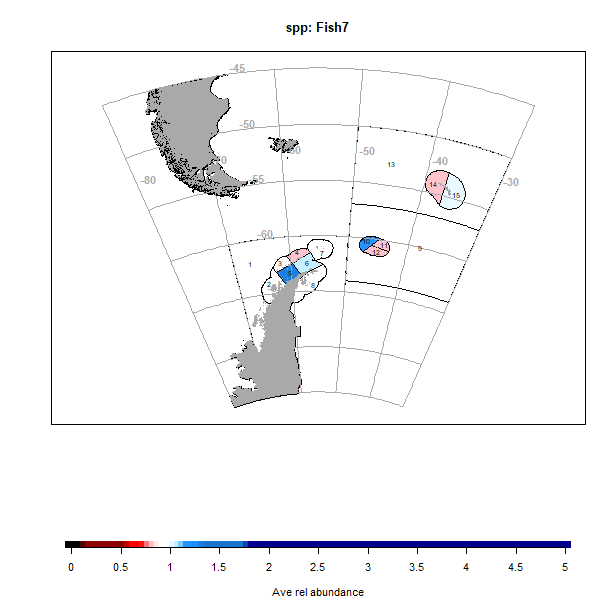

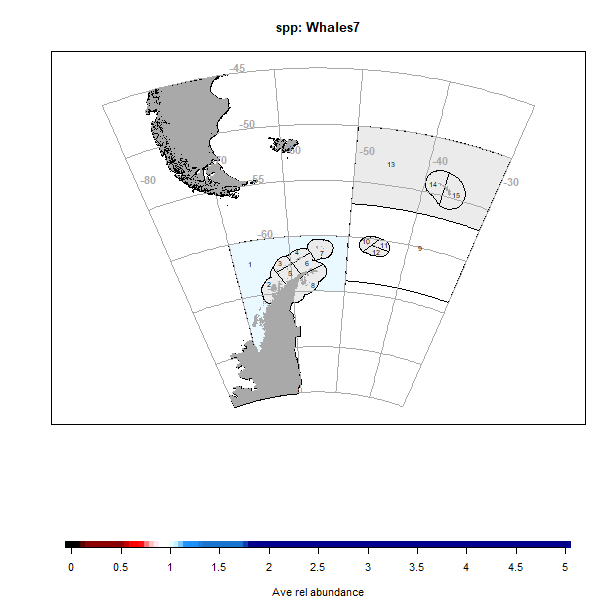


B.
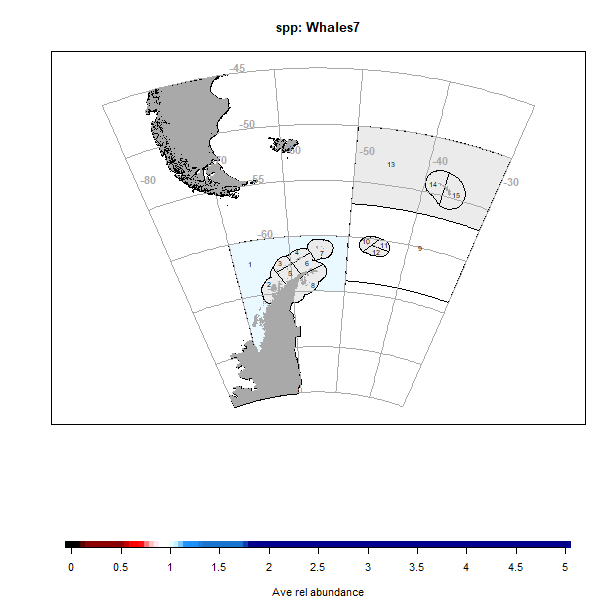


C.

D.

A.

Relative abundance, FBM/No FBM

**Fig S3.** **SSMU-specific** **outcomes of FBM-Pengs for additional predator groups under a modeled climate change impact**. Projected whale (A, B) and fish (C, D) abundances given climate-change impacts on krill growth, with outcomes at 30 years in to the model run in the left column (A, C), and at 100 years in the right (B, D). All other details as in Fig S2.


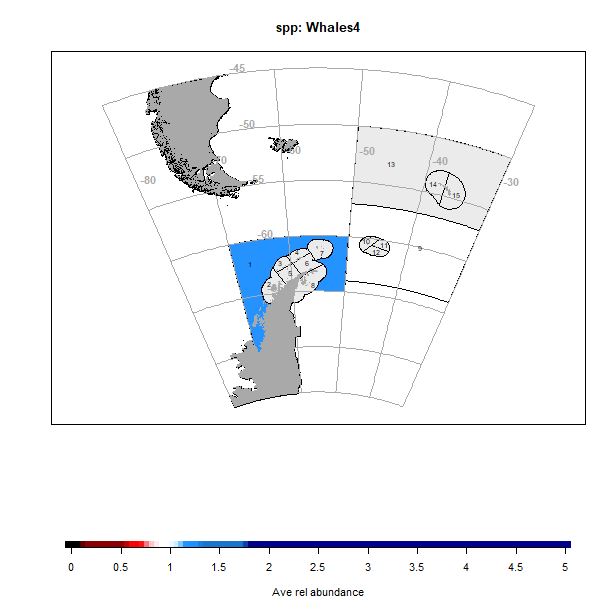

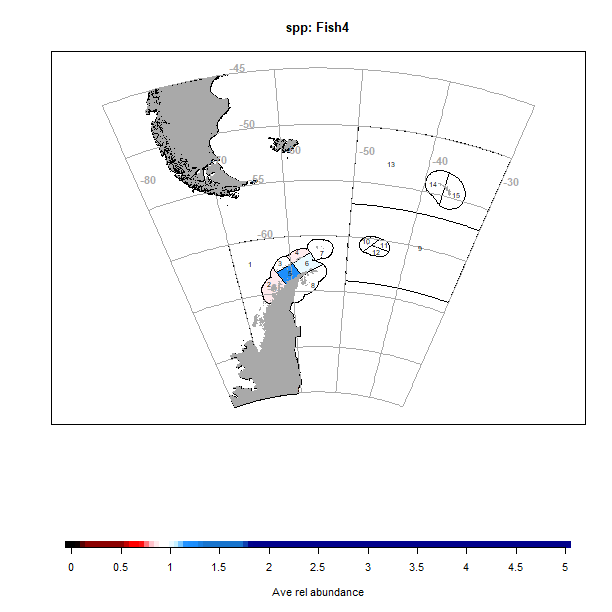

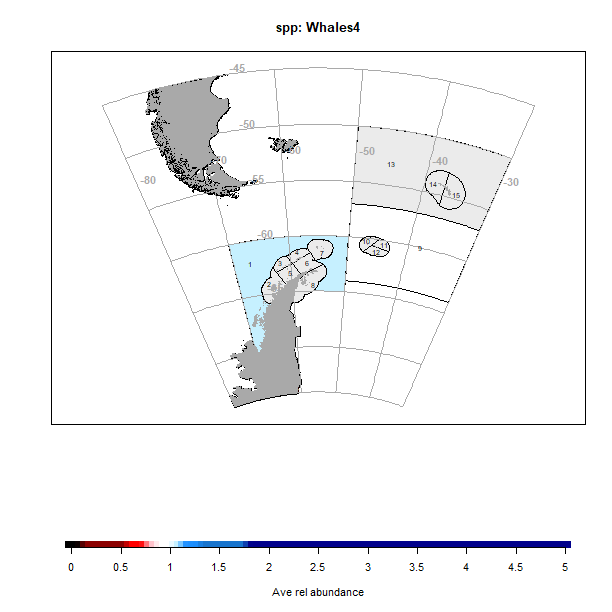

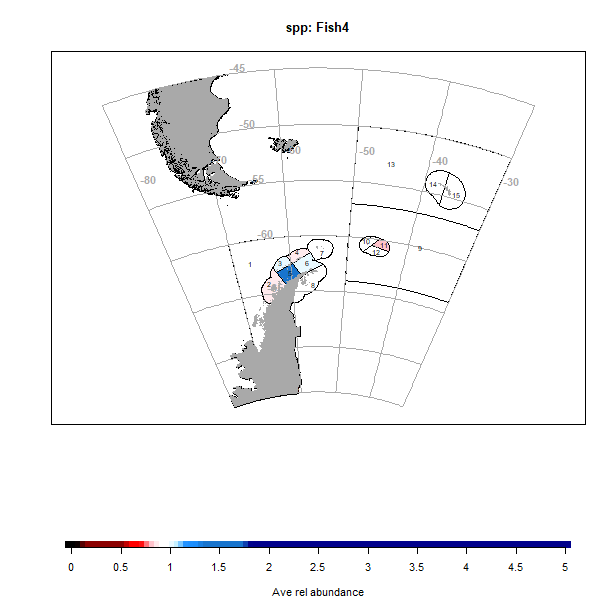


Relative abundance, MPA/NoMPA

B.

C.

D.

A.


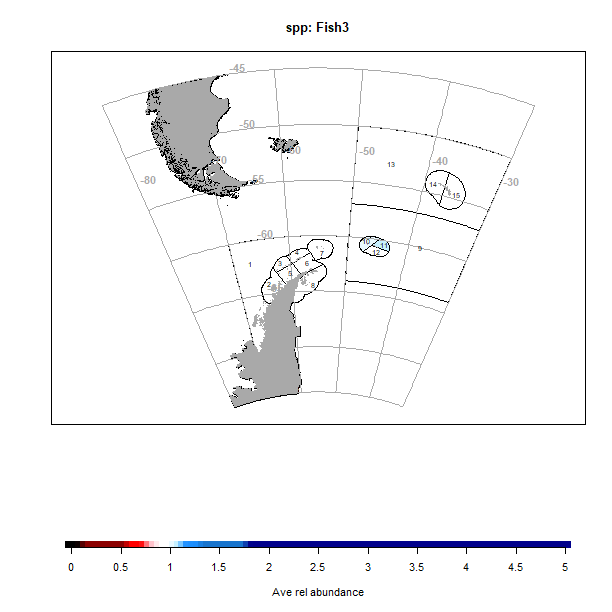


**Fig S4**. **SSMU-specific** **outcomes of the MPA for additional predator groups under a modeled climate change impact**. Projected whale (A, B) and fish (C, D) abundances given climate-change impacts on krill growth, with outcomes at 30 years in to the model run in the left column (A, C), and at 100 years in the right (B, D). All other details as in Fig S2, aside from the reference being the No MPA scenario.

100 years

30 years


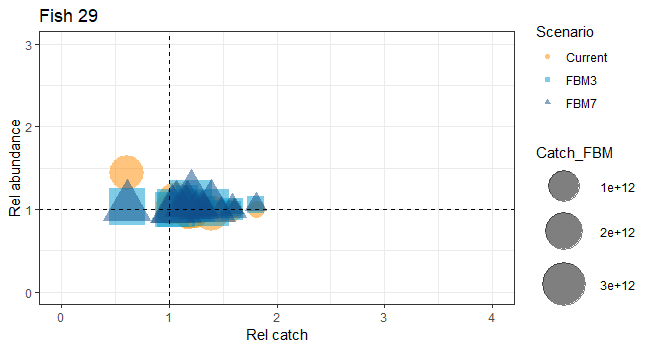

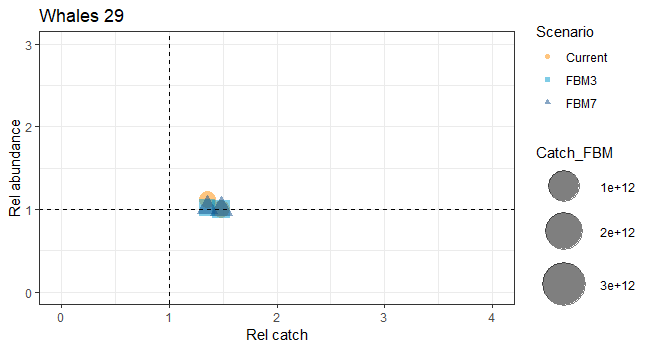


**Management scenario**

FMB-Krill

FBM-Pengs

MPA

**Catch (mT)**

1,000,000

2,000,000

B.

A.

Relative abundance

Relative catch


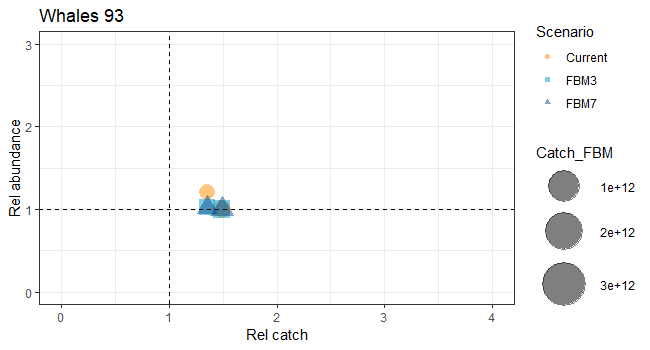

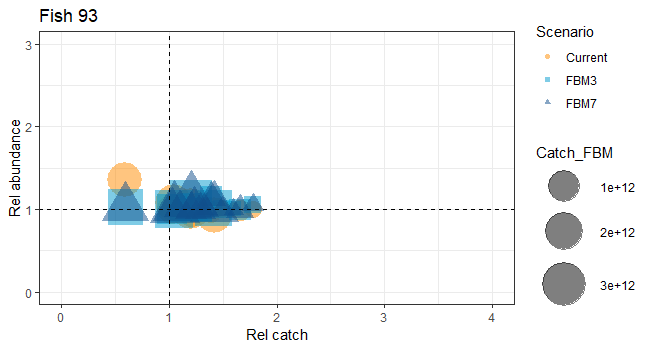


A.

B.

D.

C.

D.

C.

**Fig S5. Relationship between relative catches and the relative abundances of whales and fish given the two FBM strategies and an MPA and a modeled impact of climate change.** Relative catches (FBM/No FBM or MPA/No MPA, x-axis) and relative changes in the abundances (FBM/No FBM or MPA/No MPA, y-axis) of whales (A, B) and fish (C, D) given FBM-Krill (light blue squares), FBM-Pengs (dark blue triangles), and the MPA (orange circle) at 30 years (left column, A and C) and at 100 years (right column, B and D). The dashed lines represent no change in catch or abundance at x=1 and y=1, respectively.
