# Supplementary material for: Comparing feedback and spatial approaches to advance ecosystem-based fisheries management in a changing Antarctic
Source: PLoS One. 2020 Sep 8;15(9):e0231954. doi: 10.1371/journal.pone.0231954 (PMC7478840; doi:10.1371/journal.pone.0231954)
Supplement: Attachment — Submitted filename: pone-D-19-22435.pdf [file pone.0231954.s008.pdf]

Comparing feedback and spatial approaches to advance ecosystem-based fisheries management in a changing Antarctic

Klein and Watters

PLoS One PONE-D-19-22435

General Comments

The authors motivate an interesting and timely comparison of feedback management of catch limits versus management via marine protected area on the ecosystem-based management of the krill fishery around the Antarctic Peninsula and in the Scotia Sea. Their approach makes use of a minimally realistic ecosystem model and considers the potential effect of climate change on krill growth rates. Broader ecosystem considerations take the form of presumably generic krill predator groups (penguins, seals, whales and fish) whose dynamics respond to change in krill biomass over time and in different model subregions. It is impossible to ascertain how these groups differ from one another because relevant details on model process equations, assumptions, and/or parameter values for these different groups are almost entirely lacking. This is but one example of my essential problem with the manuscript as it stands; there is simply not sufficient detail provided to fully understand how the model is constructed, how it is parameterized and what krill & predator biology and foraging ecology can be assumed unimportant. Without this essential context, it becomes difficult to objectively judge the value of the results presented and conclusions drawn. I appreciate the authors cite a number of preceding papers that use the same/similar model but I don't have access to those currently and I don't believe that I or, more importantly, PLoS One readers should be expected to go hunting elsewhere to understand essential details of the methods used in this manuscript. Please, see the points under Specific Comments for a sense of what and where details appear to be missing.

I suggest the authors revise the Methods to focus on a description of the relevant biology that informs the krill and predator dynamics in the model. This should include equations (where helpful; as the authors have for other model components) so that the model structure is presented as unambiguously as possible along with clear and (relatively) jargon-free descriptions so that readers less familiar with the math can still follow the underlying logic. The authors should also include a table describing the key model parameters and their values and showing how particular parameters vary across the modelling scenarios. Some mitigating points regarding likely implications of certain simplifying assumptions can be made in this section but I think it helpful that at least a paragraph in the Discussion be devoted to a broader conversation about the model assumptions with respect to the underlying biology and ecology.

The authors have done an excellent, even-handed job of discussing the model results with respect to the management strategies considered. Adding the relevant details as described above, will allow the broad readership of PLoS One, most of whom will not be fisheries or ecosystem modellers, to fully appreciate the manuscript's conclusions. I also suspect that doing so will confer a bit more heft to the management advice for CCAMLR, if delivery of that advice hinges on publication of this study.

Specific Comments - by line number or section

1. l 71-72: written this way, there is an assumption that climate change will happen in the future but not now… suggest rephrasing to something like "…the need to mitigate current and future climate change effects…"
2. l 86: as above, perhaps add "...ongoing and future climate change..."?
3. l 102-103: It would be helpful for the many readers who will be less familiar with the background context to spell out the aims of Article II, succinctly, here rather than direct readers to other papers.
4. l 125: Fig 1 legend - For review purposes, figure legends should be placed together just prior to the figures, or (preferably) on the same page as the figures. It is rather inconvenient to have to flip repeatedly between text and figure pages to interpret what is going on…
5. l 140: you only seem to mention penguins throughout, are seals generically lumped in with penguins? If so, this calls into question why you make any distinction between vertebrate predator groups. There is no explanation that I can find for how penguins and seals are made distinct in

the model. Indeed, even the model name is FBM-Pengs, there is no FBM-Seals analogue? Are the different parameter values for these groups? If so, what are the parameter(s) and how do they differ between the groups? What underlying information is used to develop these parameters? All these details seem to be missing, yet are clearly important for understanding what the model is doing and for judging the value of the model results.

6. l 141-143: First, a sentence or two is warranted on the applicability of a delay-difference model for vertebrate predator dynamics. Second, what information is used to determine how far different predators forage away from breeding sites. How is this adjusted to reflect differences in at-sea distributions during breeding and non-breeding periods? Are there species-specific rules or is homogeneity assumed within groups? Many of these issues may be irrelevant, given the particular structure and assumptions of your model but as you have not described what these are or why they are imposed you have left me unconstrained to question...

7. l 148-150: Ok, you consider different kinds/rates of krill movements between subregions but what about the predator movements and/or foraging distributions? these will differ both within and between the broad groups you consider.

8. l 160: for the many readers not from a fisheries background, define "fully developed"

9. l 218-233: This is all good but you don't describe the process by which penguin abundance varies between time increments in the model. This would seem to be critical for appreciating how krill biomass and removals via fishing actually impact penguin abundance. Also, what about seals, do they follow the same dynamics just with different parameters? What are those parameters and their values? This needs to be stated clearly.
