# Supplementary material for: Comparing feedback and spatial approaches to advance ecosystem-based fisheries management in a changing Antarctic
Source: PLoS One. 2020 Sep 8;15(9):e0231954. doi: 10.1371/journal.pone.0231954 (PMC7478840; doi:10.1371/journal.pone.0231954)
Supplement: Attachment — Submitted filename: Klein_Watters_Response_to_Reviewers.docx [file pone.0231954.s009.docx]

Comparing feedback and spatial approaches to advance ecosystem-based fisheries management in a changing Antarctic

*Short title: Comparing feedback management and a marine protected area in the Antarctic.*

Emily S. Klein and George M. Watters

# Response to reviewer comments

We thank the editor and the two reviewers for their comments on our initial submission of this manuscript. Overall, the comments have provided insightful and valuable feedback that has improved the manuscript, and we hope we have addressed their concerns. We have edited the manuscript to address concerns, and our specific responses are below.

## Reviewer #1:

**Comment**: Before reading the whole article, I kind of felt after reading the abstract that the FBM strategies (FBM-Krill) provide greater benefits to predators than the MPA strategy (lines 44 – 48), yet after reading the article I know that’s not the case (this should be made clearer). In fact, the results indicate that MPAs seem to be a better strategy for both predators (except seals) and fisheries – they maintain (slightly increase) catch and increase predator abundance. Of course, it’s not as black and white as this (especially when considering increased fishing in areas of low krill density), but the tone of the article (abstract/results/discuss) comes across as slightly ‘pro-FBM’ and slightly downplaying MPAs, and the authors should consider whether the article needs a slight reframing to be more balanced.

This is particularly important where the overall benefits to penguin abundance under MPAs are the only substantial benefits from any of the three strategies to either biodiversity or fisheries and it should be stated more clearly. Particularly when you determine that total catch was only maintained by the FBM strategies and only slightly increased with MPAs – which starts to raise the question about whether fisheries and fisheries managers would really be interested in applying such strategies if there are also no obvious substantial outcomes for predators (such as the two FBM strats)? It costs time and resources to apply a strategy, so the outcomes/benefits need to be tangible and substantial for industry or CCAMLR to take note (thus it is important to highlight the benefits provided under any strategy).

***Response****: We thank this reviewer for their time and effort in reviewing our work, and their commitment to improving the manuscript. Their feedback has improved the work, and we hope we have adequately addressed their concerns.*

*We have addressed the Abstract in response to this comment, and updated results reported there.*

*Indeed, in general there were overall benefits to some predators the MPA and little or no benefits overall with either FBM (Fig 2), but different patterns emerge at smaller spatial scales (Figs 3-5 and S2-S4) – where some areas did report modest increases under FBM. Regardless, the point this reviewer makes that applying any of these strategies takes time and resources is an important one, and we have worked to consider this more fully throughout the manuscript. We do note that, in addition, the work is more to provide advice to CCAMLR on actions it is already undertaking, rather than convince CCAMLR to do something new. We have adjusted some text to this end as well.*

**Comment:** What kind of seals are you talking about – are they ice-dependant or ice independent? And are you including all 4 Antarctic penguin species?

***Response:*** *We have recreated table B1 from Watters et al. 2013 to address this comment.*

**Comment:** It would probably be helpful having a flow chart detailing the important steps/variables in the methods (e.g. number of simulations, main indicators, names of the scenarios, timeframe, what the output variable being measured is etc). This would help the reader follow the process.

***Response:*** *We have created a new figure, now Fig 2, to address this comment*.

**Comment**: You didn’t undertake any statistical analysis to determine how much each of the strategies differed from the baseline (No FBM/No MPA), and instead use terms like ‘minimal increases’ etc, which is fine – though it would be useful to provide a scale for the reader to determine what a substantial impact on predator abundance/ fisheries catch is. For example, Fig 2 shows a ~0.35 increase in penguin abundance under the MPA scenario (for 2100) – is this equivalent to a 35% increase in abundance? Which certainly appears to be a substantial increase. If a 35% increase is the wrong interpretation, the scale needs to be clarified as this is how it will be interpreted. You could also consider undertaking statistical analyses to compare the strategies to the baselines (ANOVAs would also allow you to compare between scenarios).

***Response:*** *Yes, it is a 35% increase. To further respond to this comment and help clarify results given this concern, we implemented a very general scale for comparing results. However, this scale has little ecological or biological relevance, as the importance of changes in populations of animals depends strongly on the species at hand and their life history. For example, a small percent increase in a long-lived species, such as whales, may be significant to that population, but the same percent increase may be less significant to another species. Further, whether a percent difference in a population or a fishery is significant may also depend on the reader – e.g. a percent change in the fishery may seem unimportant to some, but be very important to others. Moreover, we believe ecosystem models should be used as strategic and not tactical advice – that is, attaching “significance” to specific numbers in our results may infer tactical outcomes. Instead, we encourage readers and other stakeholders to consider our findings in strategic ways forward instead of as precise answers. For these reasons, we chose not to undertake further assessment of our outcomes beyond the scale we now introduce, and have added text on this point.*

**Comment**: Data availability – it might be helpful for managers and conservationists to have access to the spatial data layers of the SSMU outcomes so that they can utilise them in spatial planning or future studies.

***Response:*** *The layers are simply plotting findings across the SSMU so they are more readily understandable. We have included the numbers behind these maps in a new Supporting Info spreadsheet, where the recovery of these numbers is simply taking the FBM or MPA result and dividing by the No FBM or No MPA result – but managers, conservationists, and others are welcome to use the numbers in any maps as they see fit.*

**Comment**: Line 46 – make it ‘outcomes of our second FBM option’ to make it clearer that you are referring to the FBM option rather than the second overall options that you mention in line 40. Maybe reword the line 40 one as ‘strategies’ to keep it consistent with the rest of the article?

***Response:*** *We have made these changes, lines 41 and 46.*

**Comment**: Lines 61-65 – Some of these statements could use references

***Response:*** *We have added several references.*

**Comment**: 68 – might be useful to give an example of observed climate impacts in the Southern Ocean rather than just referring to refs

***Response:*** *We have included examples from the references included, now line 69.*

**Comment**: 95-96 – its not really the decision makers that are robust/adapt to the change, it’s the target/ecosystem that needs to be robust or adapt

***Response:*** *We agree this was confusing – the decision makers are not necessarily able to adapt or be robust, it is the management strategy that either responds to or is robust to change. We have adjusted text to reflect this.*

**Comment**: 97-99 – needs referencing, and would be better split into two sentences.

***Response:*** *This sentence introduces these ideas, and the references and more detail are provided in the supporting sentences that follow.*

**Comment**: 101-102 – where are the FBM specific objectives/priorities stated by CCAMLR?

***Response:*** *We have edited to address this comment.*

**Comment**: 104 – what important krill fishing area?

***Response:*** *We have added in details to designate this area.*

**Comment**: 109-112 – beginning with ‘these models’ – split this sentence into 2 – 3 sentences to make it easier to read

***Response:*** *We have edited this section as suggested.*

**Comment**: 117 – should specify that krill density and penguin abundance are the indicators.

***Response:*** *We have edited this sentence.*

**Comment**: 118 – how can you project a new MPA based on previously shown ecological benefits (the ecological benefits of a proposed MPA haven’t been established yet)? This needs more explaining/ simplifying to make it less confusing

***Response:*** *We have edited this section.*

**Comment**: 123 – this is a very broad statement?

***Response:*** *Indeed, we believe there are implications of our work beyond the Southern Ocean, which we delineate in the Discussion.*

**Comment**: Fig1. There is no context to this figure in the intro, it should probably be first referenced in the methods? Subaresas of what?

***Response:*** *We have moved the figure caption (and therefore the figure) to the Methods section. The Subareas are designated by CCAMLR; we now note that in the caption.*

**Comment**: 135-138 – what does Fig. 1 have to do with this/ how does it support this sentence?

***Response:*** *The sentence references the Antarctic Peninsula and Scotian Sea, but we have taken this reviewer’s advice and moved the figure as suggested by the above and a later comment.*

**Comment**: 141 – might be worth providing some additional info on the delay-difference equations

***Response:*** *We have edited this sentence as suggested.*

**Comment**: 155 – this is where you should introduce Fig 1. Otherwise it seems out of context, have no idea what subareas area etc

***Response:*** *We have moved Figure 1 here as requested.*

**Comment**: 156-157 – management mechanism? Or ‘management relevant scale that actions can be implemented at’

***Response:*** *The use of subdiving spatial areas are a mechanism management can use to meet objectives.*

**Comment**: 157 – what do you mean by the entire model arena? (average of all models?) All 1001 trials? (which I only know about from later in the methods – needs to be explained at first mention)

***Response:*** *We have added text to clarify we mean the model spatial arena.*

**Comment**: 160 – on what time scale? Plausible future for when?

***Response:*** *The plausible future when the fishery is fully developed – there is no time scale associated with this plausibility. We have adjusted text in this section.*

**Comment**: 174 – Can you provide further justification for only using RCP8.5 (I’m not disagreeing that you shouldn’t, but further justification should be provided)

***Response:*** *We included only one RCP for simplicity, and added text on this and regarding the implications of only using this one in the discussion.*

**Comment**: 185-187 – what link does estimating the breeding abundance of penguins using remote sensing have to do with developing a FBM? This needs to be made clearer to better justify the claim that both strategies have interest and support from various stakeholders.

***Response:*** *We have included text to clarity this point.*

**Comment**: 188 – what are some of the alternative indicators/ possible approaches?

***Response:*** *We included text on this point.*

**Comment**: 248 – what recent fishing patterns and from where?

***Response:*** *The spatial and seasonal distributions of krill catches references in the preceding sentence. We have edited to clarify.*

**Comment**: 278 – better worded as ‘Relative abundances of krill predators were sensitive to…’?

***Response:*** *We have updated the text to this suggestion.*

**Comment**: 281 – so actively managing fisheries based on penguin abundance leads to a decline in penguins and seals (therefore managers do worse than if they did nothing) – is that realistic? Coming back to this after reading the discussion section about FBM-Pengs – you explain why the model might have produced the results it did, though do you think the model behaviour is realistic?

***Response:*** *We believe this model behavior is realistic, given the text explaining this in the discussion – i.e. that we maintained overall catch and therefore shifted fishery effort in to areas where they were likely vulnerable penguin populations, simply because these areas did not decline as much as others. This seems a reasonable, albeit undesirable and not particularly intuitive, outcome.*

**Comment**: Fig 2. When you say ‘All results are referenced to the No FBM or No MPA scenarios.’ do you mean via the dotted line? (if so that should be specified)

***Response:*** *Result are relative to the No FBM or No MPA scenario; we have updated the caption for clarity on this point.*

**Comment**: Fig 3. Might be useful to have the timeframes labelled on the figure itself. For the broad overall trends presented in Fig 2 – do these only include the average of the SSMUs, or did you model the predator abundance across the subareas too? (can you specify)

***Response:*** *Results in Fig 3 are aggregated across the entire model spatial arena – i.e. total populations or fishery outcomes with FBM or an MPA relative to the total population or fishery outcome in the No FBM or No MPA scenario - they are not averages. We have adjusted the figure caption on this, and labeled the time periods.*

**Comment**: Fig 5. Can you include the MPA’s in the figure – hard to compare results against it when it is not labelled/depicted. Also given that figures should stand alone without needing the text, you might need the full text in each figure legend (rather than referring to Fig 3) – but this probably depends on the journals standards.

***Response:*** *We aim to keep are mapped figures comparable across FBM strategies and the MPA, therefore we would prefer to avoid including additional and potentially distracting lines in Fig 5.*

**Comment**: Fig 6. Dashed line is ‘No FBM’? – you should add that info in (same comment as for Fig 2.)

***Response:*** *We have updated the caption on this point.*

**Comment**: 375 – 381 – This is better suited to the introduction. The discussion should be giving the stated insights.

***Response:*** *We have removed this section.*

**Comment**: 383 – simplify. Suggest something like ‘Decision makers must consider trade-offs in determining the best way to manage the krill industry’

***Response:*** *We believe this sentence is acceptable as it and conveys our intended meaning.*

**Comment**: 390 – not sure this is an appropriate use of the word offset, at least in a conservation sense (is the decreased penguin abundance really adequately offset by a lower probability of fishing in areas of low krill density?)

***Response:*** *We have changed our wording to address this comment.*

**Comment**: 393 – But only a single seal population declined (based on figure 5 and 6)?

***Response:*** *The total abundance of seals declined with an MPA (Fig 2); at the SSMU level, only one population increased – others declined or remained the same by the end of the model run (light reds in Fig 5D and no orange circles above the y=1 line in Fig 6D).*

**Comment**: 391 & 394 – when you are talking about areas of low krill density, are you referring to the ‘threshold violation’ results from Fig 2? This should be made clearer in both the results and the discussion, else it appears that there are no results depicting low krill density and it is hard for the reader to find support for this statement (and either way there are no spatial results indicating where fishing occurred in low krill density areas?)

***Response:*** *We have updated the text for clarification.*

**Comment**: 397 – 399 – round-about way of writing the sentence, please simplify

***Response:*** *We have edited this sentence.*

**Comment**: 401 – 403 – Suggesting that managing Krill via FBM as a conservation strategy for predators is somewhat misleading – there were only extremely minor benefit to penguins using FBM-Krill and declines in seals (Figure 2) and there was no benefit to the fishery (Fig 2 & your statement in #299). Yet it would cost money to apply FBM and update it each time, and this is resources that could go to managing predator populations in a more effective way?

***Response:*** *We* *agree, and have edited the section to clarify our meaning and address this point*

**Comment**: 405 – What trade-offs? Please specify.

***Response:*** *We believe the sentence is clear, and adding specifics would be superfluous.*

**Comment**: 409 – 411 – Good, this is an important finding

**Comment**: 440 – 445 – Good, this is a good conclusion

***Response:*** *We thank the reviewer for these confirmations - we agree!*

**Comment**: 449 – potentially reword? ‘It is widely believed that routine application …’

***Response:*** *We edited this sentence.*

**Comment**: 458 – people? Fisheries is a better term

***Response:*** *We prefer to denote that it is indeed people who would be impacted.*

**Comment**: 489 – Words like panacea and pathological can be simplified

***Response:*** *We appreciate the comment.*

**Comment**: 516 – The sentence beginning with ‘In fact’ doesn’t make sense (before CCAMLR?)

***Response:*** *We have edited for clarity.*

**Comment**: 520 – 522 – Sentence doesn’t make sense – something about the structure/position of the commas.

***Response:*** *The sentence was missing a word – we have updated it.*

**Comment**: 523 – instead of ‘steps’, something like ‘advancement toward’

***Response:*** *We edited this sentence.*

**Comment**: 34/35 – you generally can’t base statements on things that haven’t been published or are not in press yet (see lines 118/119 – I went to look at the reference because I wanted to know more)? Are these in press? (what does forthcoming mean?)

***Response:*** *We have updated these references.*

## Reviewer #2:

**Comment**: It is difficult to fully evaluate this manuscript as essential methodological detail is missing. The authors cite earlier papers in lieu of providing these details but I don't think this appropriate for basic information that is essential to understanding what the authors have done and, hence, for evaluating the results and conclusions. This is why I have answered "Partly" and "No" to questions 1 and 2.

The authors motivate an interesting and timely comparison of feedback management of catch limits versus management via marine protected area on the ecosystem-based management of the krill fishery around the Antarctic Peninsula and in the Scotia Sea. Their approach makes use of a minimally realistic ecosystem model and considers the potential effect of climate change on krill growth rates. Broader ecosystem considerations take the form of presumably generic krill predator groups (penguins, seals, whales and fish) whose dynamics respond to change in krill biomass over time and in different model subregions. It is impossible to ascertain how these groups differ from one another because relevant details on model process equations, assumptions, and/or parameter values for these different groups are almost entirely lacking. This is but one example of my essential problem with the manuscript as it stands; there is simply not sufficient detail provided to fully understand how the model is constructed, how it is parameterized and what krill & predator biology and foraging ecology can be assumed unimportant.

Without this essential context, it becomes difficult to objectively judge the value of the results presented and conclusions drawn. I appreciate the authors cite a number of preceding papers that use the same/similar model but I don’t have access to those currently and I don’t believe that I or, more importantly, PLoS One readers should be expected to go hunting elsewhere to understand essential details of the methods used in this manuscript. Please, see the points under Specific Comments for a sense of what and where details appear to be missing. I suggest the authors revise the Methods to focus on a description of the relevant biology that informs the krill and predator dynamics in the model. This should include equations (where helpful; as the authors have for other model components) so that the model structure is presented as unambiguously as possible along with clear and (relatively) jargon-free descriptions so that readers less familiar with the math can still follow the underlying logic. The authors should also include a table describing the key model parameters and their values and showing how particular parameters vary across the modelling scenarios. Some mitigating points regarding likely implications of certain simplifying assumptions can be made in this section but I think it helpful that at least a paragraph in the Discussion be devoted to a broader conversation about the model assumptions with respect to the underlying biology and ecology.

The authors have done an excellent, even-handed job of discussing the model results with respect to the management strategies considered. Adding the relevant details as described above, will allow the broad readership of PLoS One, most of whom will not be fisheries or ecosystem modellers, to fully appreciate the manuscript’s conclusions. I also suspect that doing so will confer a bit more heft to the management advice for CCAMLR, if delivery of that advice hinges on publication of this study.

***Response:*** *We thank this reviewer for their comments and support of our work, and understand their concerns. We agree that all of these model elements must be available and transparent – however, we respectfully disagree that this level of detail needs to be included here, for several reasons.*

*First, including all of these elements would mean extensive additional content be added to this manuscript - for example, the previously published description of model equations alone is 12 pages in length (Watters et al. 2013, Appendix A). Indeed, the information requested makes up the majority of content in a paper already in the peer-reviewed literature, Watters et al. (2013). Given this, we argue it is unnecessary to include that volume of work here, and further wonder if it would be appropriate as it would be the bulk of an article published in another journal. Finally, we agree with the reviewer that readers of PLOS ONE may not be modelers, but we feel they would be uninterested in that level of detail but, given citations, would know where they could find it if they wanted to know more.*

*This leads us to our second point. The fundamental structure of the published literature is by definition about building on and citing other work. The purpose of citations is to allow readers to find previous papers, and to avoid having to re-iterate previously published work and possibly gain permission for that to be re-printed. Further, this also allows the reader to trust that, when research builds on previously published work, that work is also vetted by peer review. Collectively, this is critical for ensuring research moves forward and published manuscripts are focused on new insights. More specifically regarding ecosystem models, when authors use published models to answer new questions, it is typical for those authors to cite previously peer-reviewed papers instead of re-publishing model details (e.g. Megrey et al. 2007, Kaplan et al. 2013, Ihde and Townsend 2017).*

*Our third point makes this particularly clear in this case: one of the previous papers we cite is already published in PLOS ONE (Klein et al. 2018). That paper does not include the level of detail that this reviewer is asking for. The reviewers and editor on that manuscript accepted the citation of our previous work, and trusted the model had been well-vetted by their peers and could be found in that citation by interested PLOS ONE readers.*

*We do appreciate this reviewer’s concerns, but respectfully disagree with their requests on these grounds. However, to try and ease this concern, we have added additional information (including a new figure and table in response to this and the previous reviewer), worked to address the specific comments below, and have clarified in the Methods that the underlying model itself has not been altered from that already vetted by peer review and available in the published literature as cited in this manuscript. Finally, we also have uploaded all of our model code and input information to https://github.com/EmilyKlein/KPFM2.*

1. l 71-72: written this way, there is an assumption that climate change will happen in the future but not now… suggest rephrasing to something like “…the need to mitigate current and future climate change effects…”

2. l 86: as above, perhaps add ”...ongoing and future climate change...”?

***Response:*** *We agree with these important points and have edited these sentence as suggested.*

3. l 102-103: It would be helpful for the many readers who will be less familiar with the background context to spell out the aims of Article II, succinctly, here rather than direct readers to other papers.

***Response:*** *We edited this sentence to provide the overall aim of Article II.*

4. l 125: Fig 1 legend - For review purposes, figure legends should be placed together just prior to the figures, or (preferably) on the same page as the figures. It is rather inconvenient to have to flip repeatedly between text and figure pages to interpret what is going on…

***Response:*** *We have moved the figure legend in response to this and comments made by Reviewer 1.*

5. l 140: you only seem to mention penguins throughout, are seals generically lumped in with penguins? If so, this calls into question why you make any distinction between vertebrate predator groups. There is no explanation that I can find for how penguins and seals are made distinct in the model. Indeed, even the model name is FBM-Pengs, there is no FBM-Seals analogue? Are the different parameter values for these groups? If so, what are the parameter(s) and how do they differ between the groups? What underlying information is used to develop these parameters? All these details seem to be missing, yet are clearly important for understanding what the model is doing and for judging the value of the model results.

***Response:*** *Penguins and seals are implemented and parameterized separately in the model, as indicated by the model description in the Methods, and outline by a new Table 1. The FBM-Pengs strategy was so named because it is based on number of breeding penguins, due to interest in using penguins as an indicator species as denoted in the Methods, line 209-211. We note that there are many other indicators; certainly seals could be used as one in the future.*

*The details on how these groups are parameterized is substantial and can be found in the published paper describing this process (Watters et al. 2013 and its appendices). As discussed above, we argue it is reasonable and conventional to assume readers will refer to that paper for those details if they are interested.*

6. l 141-143: First, a sentence or two is warranted on the applicability of a delay-difference model for vertebrate predator dynamics. Second, what information is used to determine how far different predators forage away from breeding sites. How is this adjusted to reflect differences in at-sea distributions during breeding and non-breeding periods? Are there species-specific rules or is homogeneity assumed within groups? Many of these issues may be irrelevant, given the particular structure and assumptions of your model but as you have not described what these are or why they are imposed you have left me unconstrained to question...

7. l 148-150: Ok, you consider different kinds/rates of krill movements between subregions but what about the predator movements and/or foraging distributions? these will differ both within and between the broad groups you consider.

***Response:*** *We have included detail in the Methods to address these questions.*

8. l 160: for the many readers not from a fisheries background, define “fully developed”

***Response:*** *We have included detail here for clarification.*

9. l 218-233: This is all good but you don’t describe the process by which penguin abundance varies between time increments in the model. This would seem to be critical for appreciating how krill biomass and removals via fishing actually impact penguin abundance. Also, what about seals, do they follow the same dynamics just with different parameters? What are those parameters and their values? This needs to be stated clearly.

***Response:*** *We have added some additional detail to the Methods, and we again refer to our earlier statement.*

**References related to our response**

1. Watters GM, Hill SL, Hinke JT, Matthews J, Reid K. Decision-making for ecosystem-based management: evaluating options for a krill fishery with an ecosystem dynamics model. Ecol Appl. 2013;23(4):710-25.
2. Megrey BA, Rose KA, Ito S, Hay DE, Werner FE, Yamanaka Y, Aita MN. North Pacific basin-scale differences in lower and higher trophic level marine ecosystem responses to climate impacts using a nutrient-phytoplankton–zooplankton model coupled to a fish bioenergetics model. Ecol Model. 2007;202:196-210. doi:10.1016/j.ecolmodel.2006.08.018.
3. Kaplan IC, Brown CJ, Fulton EA, Gray IA, Field JC, Smith ADM. Impacts of depleting forage species in the California Current. Environ Conserv. 2013;40(4):380-93. Epub 04/10. doi:10.1017/S0376892913000052.
4. Ihde TF, Townsend HM. Accounting for multiple stressors influencing living marine resources in a complex estuarine ecosystem using an Atlantis model. Ecol Model. 2017;365:1-9. doi:10.1016/j.ecolmodel.2017.09.010.
5. Klein ES, Hill SL, Hinke JT, Phillips T, Watters GM. Impacts of rising sea temperature on krill increase risks for predators in the Scotia Sea. PLoS One. 2018;13(1):e0191011.
