# Supplementary material for: Comparing feedback and spatial approaches to advance ecosystem-based fisheries management in a changing Antarctic
Source: PLoS One. 2020 Sep 8;15(9):e0231954. doi: 10.1371/journal.pone.0231954 (PMC7478840; doi:10.1371/journal.pone.0231954)
Supplement: Attachment — Submitted filename: Klein_Watters_Response_to_Reviewers.docx [file pone.0231954.s010.docx]

Comparing feedback and spatial approaches to advance ecosystem-based fisheries management in a changing Antarctic

*Short title: Comparing feedback management and a marine protected area in the Antarctic.*

Emily S. Klein and George M. Watters

# Response to reviewer comments

We thank the editor and the reviewer for their continued efforts with our work, and we hope we have addressed the final needs on this paper. We appreciate their time and support.

## Reviewer #1:

**Comment**: I appreciate the Authors' detailed response to my review and generally agree with their fundamental points regarding: 1) the length of additional context required to fully describe their previously published model; and 2) the citation of previous studies to support deriving studies/publications. Regarding point 2, I would add that any published study should be sufficiently self-contained that readers (and reviewers) can understand its methodology, at least conceptually, and therefore be able to judge the value of the results and conclusions drawn. I found some methodological context lacking in the original manuscript, and, as I stated in my original review, did not have access at that time to the preceding publications in order to better understand the model structure. In situations where essential aspects of a submitted paper's methodology are published elsewhere, Authors should provide the additional publication(s) as supporting documents to aid review and/or aggregate and condense those details in Supplementary Information. Both of these practices are commonplace and a courtesy to reviewers and interested readers by not placing upon them the burden of searching for the information across (potentially) multiple sources. Providing such detail as Supporting Information also enhances a study's reproducibility, which is also a fundamental element of publication (see my comment on line 168, below). Citation of the preceding studies, as the Authors stated, serves to assure readers that the foundational work has been vetted by peer review.

I do realise this approach places an extra burden on getting work published, at times I have grudgingly laboured under this burden myself, but it generally enhances readership and citation of numerically intensive studies.

Upon reading both the Author's Response to my original review and their revised manuscript, I am satisfied that most of the issues I originally raised have been addressed. Figure 2 nicely provides more context on the overall approach, and the discussion of model assumptions is a welcome addition.

***Response****: We again thank this reviewer for their time and for continuing to engage with us to improve this manuscript. We also agree with their points here, and the importance of manuscripts standing alone – the discussion here illuminates, at least to us, a challenge in publication that should be more deeply considered, and we thank this Reviewer for urging us to do so. We have added additional text to the manuscript and Supporting Information, as well as two tables and READ ME files to the SI. We are hopeful this will work; if additional details are necessary, we ask for more specific instruction on what would be useful. We sincerely wish to adequately address these concerns but it remains somewhat unclear to us what is needed as well as also possible without recreating the previously published text of Watters et al. (2013).*

**Comment:** A fairly minor point: given the two FBM strategies considered rely on updating catch limits based on changes in either krill density or penguin abundance, I still find it confusing that the Authors state that they "...focus on penguins and seals to simplify results..." (lines 152-153). I understand that the ecosystem model includes all the major krill predator groups but I can find no prior rationale why seals need be considered in these results when the FBM strategies are based on either krill or penguin indicators. Presumably, the predicted relative abundance of seals under the differing strategies provides a comparison to penguins, thereby giving a sense of how each strategy may benefit (or not) the broader ecosystem. An extra sentence in the last paragraph of the Introduction is probably all that is needed to clarify.

***Response:*** *We find this comment quite important, and critical that we clarify. First, the Reviewer is correct in their assessment: indicators are useful as they can simplify management objectives and approach, but there will still be larger, ecosystem outcomes of changes in management beyond the indicator species (of course). Here, outcomes for seals allow us some limited insight into how using one indicator species may have implications for others – i.e. comparing consequences for seals to those for penguins, as the Reviewer surmises. Second, our reasoning for seals and penguins in the main text and having other krill predators in Supporting is not to say these other species are less important or are not also a window into impacts on additional outcomes, but to maintain a manageable set of results in the text, given our figures are already extensive with two species – i.e. that is a logistical consideration only.*

*It is important these points are clear for readers, and we have addressed the text to hopefully make them so (starting line 142).*

**Comment:** Figure 1 - it would be helpful to label the SSMU's 1-15, even if a smaller font is required. Doing so would then give spatial context to Table 1, which could be pointed out in the caption to Table 1.

***Response:*** *We have altered Figure 1, increasing the map size and using red to help the SSMU numbers stand out. We have also noted it in the caption for Table 1 as helpfully suggested.*

**Comment:** Line 163 - have these data been published? if so, would be useful to cite.

***Response:*** *We believe this comment refers to the reference set of four parameterization, which is indeed published and the citation is referenced (Watters et al., reference 39 in the main text).*

**Comment:** Line 168 - I am pleased to see the code is now provided in a GitHub repository with a detailed description and analysis guide for readers interested in reproducing or extending the Authors' work. The Authors probably did not intend this but it's useful to point out that while such code repositories are enormously helpful for reproducibility, they are not a replacement for a sufficient explanation of a study's methodology.

***Response:*** *We agree, and have added additional text in the main manuscript and Supporting Information to this effect.*
